# Surrogate measures of first-phase insulin secretion versus reference methods intravenous glucose tolerance test and hyperglycemic clamp: a systematic review and meta-analyses

**DOI:** 10.1136/bmjdrc-2024-004256

**Published:** 2024-07-16

**Authors:** Rebecka Renklint, Youssef Chninou, Martin Heni, Andreas Fritsche, Hans-Ulrich Haering, Robert Wagner, Julia Otten

**Affiliations:** 1 Department of Public Health and Clinical Medicine, Umea University, Umea, Sweden; 2 German Center for Diabetes Research (DZD), Neuherberg, Germany; 3 Department of Internal Medicine IV, Division of Diabetology, Endocrinology and Nephrology, Eberhard-Karls University Tübingen, Tübingen, Germany; 4 Department for Diagnostic Laboratory Medicine, Institute for Clinical Chemistry and Pathobiochemistry, University Hospital of Tübingen, Tübingen, Germany; 5 Institute for Diabetes Research and Metabolic Diseases, Helmholtz Center Munich, University of Tübingen, Tübingen, Germany; 6 Leibniz Center for Diabetes Research at Heinrich-Heine-University Düsseldorf, German Diabetes Center, Düsseldorf, Germany; 7 Department of Endocrinology and Diabetology, University Hospital Düsseldorf, Heinrich-Heine-University Düsseldorf, Düsseldorf, Germany

**Keywords:** Insulin Secretion

## Abstract

**Introduction:**

In this systematic review, we investigated the diagnostic accuracy of surrogate measures of insulin secretion based on fasting samples and the oral glucose tolerance test (OGTT). The first phase of insulin secretion was calculated using two gold standard methods; the hyperglycemic clamp (HGC) test and intravenous glucose tolerance test (IVGTT).

**Research design and methods:**

We conducted searches in the PubMed, Cochrane Central, and Web of Science databases, the last of which was conducted at the end of June 2021. Studies were included that measured first-phase insulin secretion in adults using both a gold-standard reference method (either HGC or IVGTT) and one or more surrogate measures from either fasting samples, OGTT or a meal-tolerance test. QUADAS-2, a revised tool for the quality assessment of diagnostic accuracy studies, was used for quality assessment. Random-effects meta-analyses were performed to examine the correlation between first-phase measured with gold standard and surrogate methods.

**Results:**

A total of 33 articles, encompassing 5362 individuals with normal glucose tolerance, pre-diabetes or type 2 diabetes, were included in our systematic review. Homeostatic model assessment (HOMA)-beta and Insulinogenic Index 30 (IGI(30)) were the surrogate measures validated in the largest number of studies (17 and 13, respectively). HOMA-beta’s pooled correlation to the reference methods was 0.48 (95% CI 0.40 to 0.56) The pooled correlation of IGI to the reference methods was 0.61 (95% CI 0.54 to 0.68). The surrogate measures with the highest correlation to the reference methods were Kadowaki (0.67 (95% CI 0.61 to 0.73)) and Stumvoll’s first-phase secretion (0.65 (95% CI 0.58 to 0.71)), both calculated from an OGTT.

**Conclusions:**

Surrogate measures from the first 30 min of an OGTT capture the first phase of insulin secretion and are a good choice for epidemiological studies. HOMA-beta has a moderate correlation to the reference methods but is not a measure of the first phase specifically.

**PROSPERO registration number:**

The meta-analysis was registered at PROSPERO (Id: CRD42020169064) before inclusion started.

**WHAT IS ALREADY KNOWN ON THIS TOPIC**

- Surrogate indices of insulin secretion are used in epidemiological studies. Until now, surrogate measures of first-phase insulin secretion have not been compared and validated in a meta-analysis.

**WHAT THIS STUDY ADDS**

- The key question of this meta-analysis is which surrogate indices are most suitable for the measurement of first-phase insulin secretion when gold-standard measurements cannot be used.

**HOW THIS STUDY MIGHT AFFECT RESEARCH, PRACTICE OR POLICY**

- This systematic review guides clinical practice when choosing a surrogate index to estimate first-phase insulin secretion in epidemiological studies and clinical settings.

## Introduction

 The global burden of diabetes was estimated to be 463 million people in 2019 and is steadily rising.^1^ The key pathomechanisms of type 2 diabetes are impaired insulin sensitivity combined with a progressive loss of pancreatic beta-cell function. Longitudinal studies have shown that beta-cell function is altered many years before the diabetes diagnosis.^2^ With the increasing recognition of disease heterogeneity, insulin secretion has been used as a key feature in the subclassification of pre-diabetes and diabetes.^3^ In one recent work, adult-onset diabetes has been classified into five clusters, each of which features different pathophysiologic phenotypes and distinct patterns of complication.^4^ In this approach, a reliable measurement of insulin secretion is important to differentiate insulinopenic and hyperinsulinemic diabetes endotypes. In pre-diabetes subphenotyping, adequate methods for measurement of insulin secretion are similarly important for capturing key differences between pre-diabetes subphenotypes.

In healthy individuals, insulin is secreted in a biphasic manner in response to an increase in arterial glucose concentration. The first phase lasts approximately 10 min, and the second phase reaches a plateau after 2–3 hours, provided glucose is continuously elevated, in an experimental setting.^5^ In type 2 diabetes, the first phase of insulin secretion is impaired or even absent and the second phase is decreased.^6^ The first phase of insulin secretion is frequently used in pathophysiological studies of type 2 diabetes and the prediction of type 1 diabetes.^7^ Homeostatic model assessment and beta cell function (HOMA-beta) is used to estimate insulin secretion for the classification of diabetes and can have importance in clinical settings for optimizing diabetes treatment.

There are numerous methods to assess first-phase secretion; of these the hyperglycemic clamp (HGC) test and intravenous glucose tolerance test (IVGTT) are referred to as the gold standards.^8^ Gold-standard methods are used in smaller populations for an accurate measurement of secretion, often in combination with an examination of insulin resistance in the same protocol. The HGC test allows assessment of both first-phase and second-phase insulin secretion, whereas the IVGTT is a reference test only for first-phase insulin secretion.

During the HGC test, a glucose bolus is given to quickly raise glucose levels to a fixed hyperglycemic target value, usually 125 mg/dL (6.9 mmol/L) above basal level.^9^ The elevated plasma glucose concentration is then maintained by continuous glucose infusion. Blood samples are taken frequently using a catheter to measure blood glucose, and the glucose infusion is adjusted as necessary. The first phase of insulin secretion is calculated from insulin levels during the first 8–10 min, and the second phase is calculated as the mean insulin value after the initial secretion peak.^9^

When performing an IVGTT, only one glucose bolus is given intravenously, and blood samples are collected for 3–4 hours. The most commonly used parameter is acute insulin response (AIR), which is the mean insulin concentration above basal levels during the first peak.^10^

The two reference methods are used to calculate the first phase of insulin secretion in similar ways and research settings. During the HGC test an amount of glucose, which is based on the participant’s level of glucose, is given to raise insulin secretion. In an IVGTT the bolus given is fixed. The difference between the reference methods is more evident in the second phase when an infusion of glucose is used in the HGC test. In both methods, the first phase is calculated from the insulin levels during the first minutes. Because both reference methods measure insulin secretion as a response to intravenous glucose administration, they cannot assess all aspects of physiological insulin secretion when food is consumed. Both reference methods are also time-consuming to perform. Therefore, several surrogate measures have been developed for broader use. Surrogate measures are used in a wide range of epidemiological studies wherein associations between insulin secretion and conditions related to diabetes are investigated. Since the oral glucose tolerance test (OGTT) is frequently used for the classification and diagnosis of diabetes and pre-diabetes, the additional blood samples taken to calculate a surrogate measure of insulin secretion are relatively inexpensive and time-efficient to collect.

The most frequently used surrogate measures of insulin secretion are based on the OGTT, which is performed after an overnight fast. Subjects are given an oral load of 75 mg glucose, which triggers insulin secretion.^8^ Plasma insulin or C-peptide and glucose levels are measured at baseline and repeatedly for at least 120 min.

HOMA-beta is based on a fasting blood sample and assesses insulin secretion by calculating the ratio of insulin concentration to glucose concentration minus 3.5 mmol/L.^8^

Although there are numerous surrogate indices for the first phase of insulin secretion, no systematic review has summarized the validation studies of surrogate indices. This study investigated the diagnostic accuracy of surrogate measures by assessing the correlation between first-phase insulin secretion calculated using surrogate methods and the reference methods IVGTT and HGC. This was undertaken to help researchers decide which surrogate measure to use for larger studies.

## Method

We performed a systematic review and meta-analyses according to the Preferred Reporting Items for Systematic Reviews and Meta-Analyses guidelines, 2020.^11^ Before we started, our meta-analysis was registered at PROSPERO.

### Search strategy and study selection

We searched the PubMed database, the Cochrane Central and Web of Science. The search phrases are specified in online supplemental methods 1. The databases were searched until March 01, 2020. Studies with participants with either normal glucose tolerance (NGT), pre-diabetes and type 2 diabetes were included.

To remove duplicates of studies the program EndNote (EndNote V.20.4.1, Clarivate Analytics (US) LLC) was used. In the first step (see online supplemental figure 1), the article titles retrieved were screened by RR only. This is in accordance with the Cochrane handbook of meta-analyses which deems it sufficient that this step is performed by one person only.^12^ In the second step, the abstracts of the remaining articles were read separately by YC and RR who then discussed whether to include each in this study. When a consensus could not be reached JO read the abstract and a final decision was made. In the third step, the remaining articles were read in full, and data was extracted to an Excel file by RR and YC separately to facilitate the comparison of the information. Studies were eligible if they measured first-phase insulin secretion using both a reference method (HGC or IVGTT) and a surrogate measure in the same study population. The HGC test and IVGTT needed to be performed as described above, with frequent measurements of insulin levels during the first 10 min and a first-phase insulin secretion calculated based on the first 10 min of the procedure. The surrogate measures included were based either on the OGTT, where insulin levels were analyzed during the OGTT, or on fasting samples where glucose and insulin levels were measured for the calculation of HOMA-beta. Studies were excluded if correlation coefficients between methods were lacking.

When reading the articles in full-text, 19 articles were included and 72 other articles matched the inclusion criterion of insulin secretion being measured by both a reference method and surrogate measure but lacked a correlation coefficient for the first phase between the surrogate measure and reference method. We then departed from the protocol registered at PROSPERO in order to include additional studies. Data was requested from the first author of each study, and 14 authors responded within 2 weeks. Seven of these studies were ultimately included in the meta-analysis. Seven articles were included as a result of cross-referencing.

### Data extraction and quality assessment

We extracted the following information from the included articles: (1) Name of the first author, (2) year of publication, (3) country in which the study was performed, (4) subject category (type 2 diabetes, impaired glucose intolerance (IGT), NGT, other diseases, healthy), (5) number of study participants, (6) proportions of male and female study participants, (7) age of study participants, (8) body mass index (BMI) of study participants, (9) standard measurement how the study was performed and how insulin secretion was expressed, (10) surrogate measure how this was calculated, and how insulin secretion was expressed, (11) the method used to determine a correlation (Pearson’s r or Spearman’s ρ), and (12) the correlation coefficient between the standard and surrogate measures for both all participants and for subgroups, if presented.

To assess the risk of bias of the included articles, we used a quality-assessment tool inspired by QUADAS-2, a revised tool for the quality assessment of diagnostic accuracy studies.^13^ We used the four domains validated in QUADAS-2 to rate the risk of introducing bias. In addition, to assess differences in analytical techniques two questions were added to rate the risk of the introduction of bias by analytical technique; (1) Were the same analytical techniques used for the index tests and reference tests? (2) Could the analytical technique have introduced bias?

### Data synthesis and analysis

**Figure 1:**
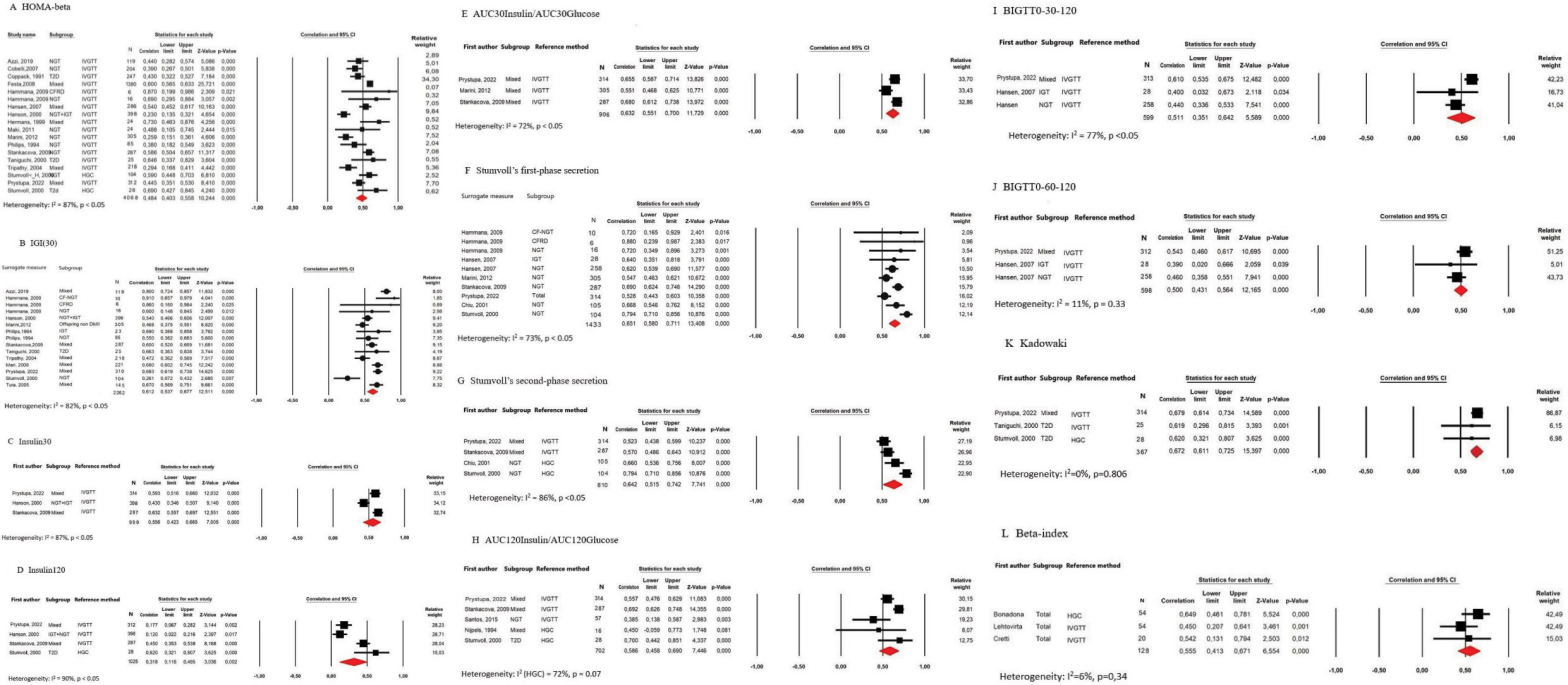
Meta-analyses of correlation coefficients between surrogate measures of insulin secretion and the reference methods intravenous glucose tolerance test (IVGTT) and the hyperglycemic clamp (HCG). CF, Cystic Fibrosis; CFRD, Cystic Fibrosis Related Diabetes; HOMA, homeostatic model assessment; IGT, impaired glucose intolerance; NGT, normal glucose tolerance; T2D, Type 2 Diabetes.

In this paper, we included all surrogate measures that have been validated against first-phase insulin secretion measured using either the HGC test or IVGTT in at least three studies (figure 1). Surrogate measures validated in two studies are also presented in the meta-analysis (online supplemental figure 2). Correlation coefficients published in only one paper are presented in online supplemental table 3. No surrogate measures from meal-tolerance tests were validated in more than two studies and these are therefore only presented in online supplemental table 3.

The main analysis, involved random-effects meta-analysis, because the characteristics of the participants differed between the studies.^14^ We also conducted fixed-effects meta-analysis as sensitivity analysis, because the sizes of the studies differed significantly and a random-effects meta-analysis may give smaller studies too much relative weight.

We used the program Comprehensive Meta-Analysis, (V.3.3 Biostat, Englewood, New Jersey, USA) which uses inverse variance weights and estimates the between-study variance τ^2^ using the DerSimonian and Laird method.^15^ The correlation coefficients are transformed to Fischer’s Z before the meta-analysis. The SE of Fischer’s Z metric is determined solely by sample size rather than by the size of the correlation, to avoid larger correlations being assigned more weight. Fischer’s Z-values are then retransformed to correlation coefficients for the graphical presentation. The risk ratio for the binary outcomes was calculated with a 95% CI and a two-sided p value.

To avoid bias we converted Spearman’s ρ to Pearson’s r according to Rupinski and Dunlap’s formula r=2sin(ρ×π/6).^16^ When the type of correlation coefficient was not specified in two studies, we contacted the first authors. When we did not receive an answer, we assumed that it was reported as Pearson’s r. Thus, all correlation coefficient presented in this article are reported as Pearson’s r.

Although all included studies presented the relationship between the reference method and the surrogate measure in terms of first-phase insulin secretion, the first phase was defined in different ways. For example, the first phase is defined as the first 5 min by Coppack *et al* and the first 3 min by Philips *et al*; however, in most studies, it is defined as the first 10 min. The methods are described in detail for each study in online supplemental table 1. We performed a separate analysis to assess the impact of different methods of calculating first-phase insulin secretion on the correlation with the surrogate measures Insulinogenic Index 30 (IGI30) and HOMA-beta (online supplemental figure 4). We also conducted meta-analyses of subgroups (NGT, IGT, and type 2 diabetes) for surrogate measurements with at least two studies with separate correlation coefficients for each subgroup (online supplemental figure 3).

### Bias estimation

We used the q-statistic to test for heterogeneity and quantified this using the I^2^ statistic. According to the interpretation of Higgins *et al*, heterogeneity I^2^ above 75% indicates that a large proportion of variance is caused by a genuine difference in the included studies.^17^ I^2^ below 25% indicates that most of the observed variance is caused by random error. To estimate publication bias, we used funnel plots with the z-plotted correlation coefficient (Fisher’s Z) on the x-axis and the SE on the y-axis, as proposed by Sterne and Egger.^18^ Funnel plots are not recommended for meta-analyses with fewer than 10 studies, but are used here as a visual aid when there were at least three studies correlating the surrogate measure to the reference method.^19^ We did not proceed with the correlation rank test because it is not recommended for small meta-analyses of fewer than 25 studies.^14^

## Results

Our initial search resulted in 12,555 titles; and 33 articles were used for the final analysis (online supplemental figure 1). Eight of the studies presented no baseline participant characteristics. The authors of these articles were contacted to retrieve information about age, sex, BMI and glycemic status; however, after a month no reply had been received from seven of these authors. Therefore, we compared the studies with missing data to the studies with complete information in a separate analysis. This is shown in online supplemental figure 5, and there was no significant difference between studies with missing data as compared with studies with complete data for HOMA-beta and IGI(30). In addition to the included articles, at one center a separate analysis was undertaken using data from two populations; 1 of 309 participants who underwent both IVGTT and OGTT and 75 participants who underwent both HGC and OGTT.^20^

The characteristics of the included studies are presented in online supplemental table 1, and the formulae of the included indices are shown in table 1. This systematic review presents surrogate measures that have been compared with first-phase insulin secretion reference methods in at least three published articles (figure 1). Surrogate measures that have been compared with first-phase insulin secretion reference methods, in two studies are presented in online supplemental figure 2. Surrogate measures that have been compared with first-phase insulin secretion reference methods in only one paper are listed in online supplemental table 2.

**Table 1:** Mathematical formulas of included surrogate indices of insulin secretion

Name of index	Formula
HOMA-beta^28^	(20 × I_0_) / (G_0_ – 3.5)
IGI(30)^29^	(I_30_ – I_0_) / (G_30_ – G_0_)
IGI(120)^29^	(I_120_ – I_0_) / (G_120_ – G_0_)
AUC30insulin/AUC30glucose^30^	Area under the curve, as calculated with the trapezoidal rule, for insulin divided by the area under the curve for glucose from 0 min to 30 min.
AUC120insulin/AUC120glucose^30^	Area under the curve, as calculated with the trapezoidal rule, for insulin divided with the area under the curve for glucose from time 0 min to 120 min.
Stumvoll’s first-phase secretion^24^	1283 + (1.829 × I_30_) – (138.7 × G_30_) + (3.772 × I_0_)
Stumvoll’s second-phase secretion^24^	287 + (0.4164 × I_30_) – (26–07 × G_30_) + (0.9226 × I_0_)
Corrected insulin response 120, CIR120^31^	I_120 _/ (G_120_ × (G_120_ – 70))
Corrected insulin response 30, CIR30^31^	I_30_ / (G_30_ × (G_30_–70))
BIGTT0-30-120^32^	exp(8.20 + (0.00178×I_0_) + (0.00168×I_30_) − (0.000383×I_120_) − (0.314×G_0_) − (0.109×G_30_) + (0.0781×G_120_) + (0.180×sex) + (0.032×BMI))
BIGTT0-60-120^32^	exp(8.19 + (0.00339×I_0_) + (0.00152×I_60_) − (0.000959×I_120_) − (0.389×G_0_) − (0.142×G_60_) + (0.164×G_120_) + (0.256×sex) + (0.038×BMI))
Kadowaki^33^	(I_30_ – I_0_) / G_30_
Beta-index^34^	A minimal model of insulin secretion applied to the glucose and C-peptide curves of each subject, as calculated by Cretti *et al*

AIR, acute insulin response; AUC, area under the curve; BIGTT, OGTT-based estimate of insulin secretion (32); BMI, body mass index; HGC, hyperglycemic clamp; IGI, Insulinogenic Index; IGT, impaired glucose tolerance; IVGTT, intravenous glucose tolerance test; NGT, normal glucose tolerance.

### Risk-of-bias assessment

The quality assessment is summarized in online supplemental table 2. In summary, 23 of the included studies were judged as low risk of bias and 10 studies as unclear risk of bias. In a sensitivity analysis, we compared studies with low a risk of bias with those with an unclear risk of bias for IGI(30) and HOMA-beta (online supplemental figure 6). The sensitivity analysis did not detect a difference between low and unclear risk of bias studies for IGI(30) and HOMA-beta (p=0.20 and p=0.45, respectively). There was no concern that the included studies did not meet the inclusion criteria for our systematic review, except for two studies; Hammana *et al*^21^ and Azzi *et al*,^22^ which studied patients with cystic fibrosis and Friedreich’s ataxia, respectively. We therefore performed a sensitivity analysis which showed no significant difference between correlations for these groups (data not shown). The studies were therefore included.

All studies used the same analytical technique for measuring glucose and insulin for both the reference and surrogate methods. In eight of the included studies, the time that passed between the performing of the reference and surrogate methods is not clearly presented; for example, in Herman *et al*^23^ the latter test was conducted “within eight weeks” of the former. Study populations were evenly distributed between sexes. There were studies that included only men or only women, but in total both sexes were equally represented. None of the studies presented separate correlation coefficients for men and women.

Publication bias was estimated using funnel plots with the SE plotted against the Z-transformed correlation coefficient (online supplemental figure 8A–M). The funnel plots of the analyses were symmetrical and did not indicate publication bias (online supplemental figure 8A).

There was significant heterogeneity (I^2^ above 50%) in the majority of the meta-analyses performed and many had I^2^ above 75%, for example, HOMA-beta (I^2^=87%), IGI(30) (I^2^=82%), Insulin30 (I^2^=87%), Insulin120 (I^2^=90%), area under the curve (AUC)120Insulin/AUC120Glucose (I^2^=72%), and OGTT-based estimate of insulin secretion (BIGTT)0-30-120 (I^2^=77%). This strengthened our decision to use random-effects instead of fixed-effects analyses, the result of which are compared in online supplemental figure 7. There was no significant difference between random and fixed effects analyses.

### Comparison of surrogate and reference measures of first-phase insulin

**Figure 2:**
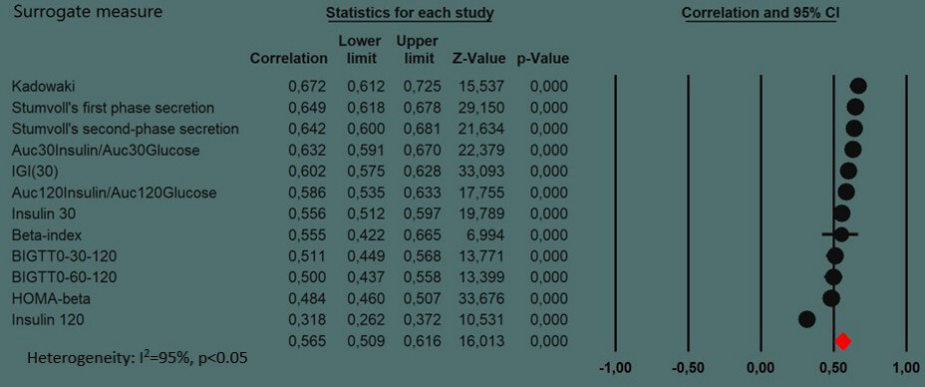
Summary of all meta-analyses of this systematic review. This figure summarizes the results of the 12 meta-analyses reported in figure 1A–L. In this figure, one row shows the results of one meta-analysis of figure 1; for example, the row “HOMA-beta” describes the results from the meta-analysis of figure 1A. The rows of this figure are ordered by the strengths of the pooled correlations, for example, the surrogate measure “Kadowaki” has the highest pooled correlation (0.672) to the gold standard examinations and the surrogate measure “Insulin 120” has the lowest pooled correlation (0.318) according to the results reported in figure 1A–L. Pooling all meta-analyses together shows that the correlations of the different surrogate measures to the gold standard examination differ substantially from each other with a heterogeneity I^2^ of 95%. AUC, area under the curve; BIGTT, oral glucose tolerance test-based estimate of insulin secretion; HOMA, homeostatic model assessment; IGI, Insulinogenic Index.

We found at least three validation studies for the surrogate measures HOMA-beta, IGI(30) Insulin30, Insulin 120, AUC30Insulin/AUC30Glucose, Stumvoll’s first-phase secretion, Stumvoll’s second-phase secretion, BIGTT0-60-120, BIGTT0-30-60, Kadowaki, AUC120Insulin/AUC120Glucose and Beta-index (figure 1). All surrogate measures validated in at least three studies are calculated from an OGTT, except HOMA-beta. The surrogate measures with the strongest correlation to reference methods were Kadowaki (0.67 (95% CI 0.61 to 0.73)) and Stumvoll’s first-phase secretion (0.65 (95% CI 0.58 to 0.71)) (figure 2). These measures were only validated in three respectively seven studies. All five surrogate measures with the highest correlation are based on the first 30 min of an OGTT. In addition, Stumvoll’s second phase is based on the first 30 min of an OGTT (table 1), and was compared with the first phase calculated using a reference method. HOMA-beta and IGI(30) were the surrogate measures validated in most studies, (17 respectively 13 studies) and their pooled correlations to reference methods are 0.48 (95% CI 0.40 to 0.56) and 0.61 (95% CI 0.54 to 0.68) respectively (figure 1A).

All meta-analyses included in figure 1 are summarized with their correlations and CIs in figure 2. The figure shows that the surrogate measure Kadowaki has the highest pooled correlation to the first phase of insulin secretion according to gold standard examinations and the surrogate measure “Insulin 120” has the lowest pooled correlation. Figure 2 shows that the correlations of the different surrogate measures to the gold standard examination differ greatly from each other which results in a heterogeneity I^2^ of 95%.

### Subgroup analysis of different calculation possibilities of reference methods

There was a difference between the calculation methods of the reference methods in correlation to both HOMA-beta (p<0.05), and to IGI(30) (p<0.05) This is also reflected in the high heterogeneity for both HOMA-beta and IGI,^24^ with an I^2^ of 87% and 81%, respectively (online supplemental figure 1A–B). For IGI(30) the difference between calculation methods can be explained for the most part by one study which differed from the rest: Stumvoll (2000). In Stumvoll’s study, the first phase was calculated as the sum of plasma insulin concentrations at 2.5, 5, 7.5, and 10 min during the HGC experiment, minus the mean basal plasma insulin concentration (online supplemental figure 4A). When that study was removed, no significant difference between the other studies remained (p<0.05). When comparing the different ways of calculating the first-phase insulin secretion reference measure (explained in online supplemental table 1), we found that the most validated calculation—AIR10min (insulin secretion during the first 10 min)—had a pooled correlation of 0.62 (95% CI 0.51 to 0.71) to IGI(30). This was similar to the correlation to other reference methods, for example, AIR08min and deltaAIR (online supplemental figure 4A). Still, the variation between the studies correlating AIR10min to IGI^24^ was large, ranging from 0.47 to 0.83.

### Subgroup analysis of NGT, pre-diabetes, and type 2 diabetes

The surrogate measures investigated in at least two different studies with correlations stratified by glycemic subgroup were HOMA-beta, IGI(30) and Stumvoll’s first-phase secretion. Analyzing these subgroups showed large differences in the correlation between studies (online supplemental figure 3). The subgroups were relatively small, the type 2 diabetes studies generally consisted of 8–36 participants, except for that of Coppack *et al*, which consisted of 247 participants. In the pre-diabetes group, the correlation between the surrogate measure (HOMA-beta (0.46 (95% CI 0.30 to 0.60)), IGI(30) (0.59 (95% CI 0.48 to 0.67)) and Stumvoll’s first-phase secretion (0.53 (95% CI 0.38 to 0.65))) and reference methods was most consistent across studies and heterogeneity was below 10% (online supplemental figure 3). We did not find any significant differences in the correlations between the different glycemic subgroups for HOMA-beta or IGI(30) and reference methods, p(HOMA-beta)=0.53; p(IGI(30))=0.86. Between Stumvoll’s first-phase secretion and reference methods, there was a significant difference between NGT (0.71 (95% CI 0.61 to 0.78)) and pre-diabetes (0.53 (95% CI 0.38 to 0.65)) (p<0.05) but only two studies had a separate correlation for pre-diabetes (online supplemental figure 3) shows the meta-analyses of the subgroups and the correlations between HOMA-beta, IGI(30) and Stumvoll’s first-phase secretion and the reference methods.

## Discussion

We performed a systematic review in order to find the most appropriate surrogate method for measuring first-phase insulin secretion, with a view to this being used in epidemiological studies. The surrogate measures with the highest correlation to the reference methods are Kadowaki and Stumvoll’s first-phase secretion. However, the most frequently validated surrogate measures are IGI(30) and HOMA-beta.

In our attempt to find a surrogate measure for the first phase of insulin secretion, we found that indices with the highest correlations to the reference methods are all based on the first 30 min of the OGTT, as is for the case of, for example, Kadowaki, Stumvoll’s first-phase secretion and IGI(30). Kadowaki is the surrogate measure with the highest correlation, but it is not as frequently used and was only validated in three studies. Like Stumvoll’s first-phase secretion, one of the surrogate measures with the highest correlation, it is based on the increment of insulin and the level of glucose after 30 min during an OGTT. Both Kadowaki and Stumvoll’s first-phase secretion are less frequently used, and fewer studies and participants validate these measures compared with IGI(30), which is similar, and had only a slightly lower pooled correlation to the gold standard reference measure.

Insulin secretion is measured mainly in research and there is no cut-off to separate healthy and diseased individuals. Although it is not used as a diagnostic tool in clinical settings an accurate measurement of insulin secretion using surrogate measures is important in order to classify individuals into different levels of diabetes development and help patients with type 2 diabetes in different clusters access more specific treatment and obtain a better understanding of the pathophysiology.

As expected, the surrogate measure with the lowest correlation to the first phase of insulin secretion is Insulin120, which is measured later in the OGTT. There are substantial differences between the reference methods and surrogate measures. With the gold standard examinations, the first phase of insulin secretion is measured during minutes after the intravenous glucose bolus. Such a direct examination of the first phase is not possible, neither with indices based on the OGTT or with those based on fasting samples. Therefore, this systematic review can only examine the association between surrogate indices and the direct measurement of the first phase. We find correlation coefficients that are moderate at the most.

However, surrogate indices after the OGTT may reflect a more physiological insulin responses. Moreover, OGTT-based surrogate measures are influenced by additional factors that do not contribute to the response to intravenous glucose. For example, the incretin effect amounts to a twofold to threefold increase in secreted insulin in response to oral glucose as compared with intravenous glucose administration.^25^ The measurements based on the OGTT could therefore provide a good estimate of physiological insulin secretion and still only having a modest correlation to the IVGTT. This applies also to measures based on a meal tolerance test; unfortunately, we had too little data to compare those in our analyses. The meal tolerance test not only mimics the uptake of glucose but also takes into account the variety of nutrients in a normal meal. The correlation to the reference method, IVGTT, could be low and the surrogate index may still be a closer estimate of physiological insulin secretion.

In type 2 diabetes the incretin effect is reduced compared with NGT individuals.^26^ This could result in substantially lower insulin secretion when measured during an OGTT. Other confounding factors are variation in gastric emptying and intestinal absorption of glucose. Variation in gastric emptying has been shown to account for 35% of the variance in peak blood glucose levels at the end of an OGTT for both healthy people and patients with type 2 diabetes. However, most of the surrogate measures involving the OGTT use the insulin-to-glucose ratio, and therefore the impact of gastric emptying should be reduced.

HOMA-beta was the surrogate measure that was validated in the most studies and is an index that captures another aspect of insulin secretion. Unlike the surrogate measure involving the OGTT, it is based on a single fasting blood sample and is not specifically intended to measure the first phase of insulin secretion. It is used as a non-specific measure of insulin secretion, without any information about an acute stimulation of beta cells and can thus be considered a measure of steady-state insulin secretion during fasting. In a recent cluster analysis aimed at reclassifying adult-onset diabetes, insulin secretion was estimated using HOMA2-beta, a computer-estimated version of HOMA, using either insulin or C-peptide in a fasting state, both of which are available in many laboratories and can be used in routine healthcare (27–31). In this systematic review, it is evident that HOMA-beta has a consistently good correlation to first-phase insulin secretion even when calculated using insulin. In addition, this correlation had the lowest heterogeneity across the analyzed studies. This encourages the use of HOMA-beta as an estimate of insulin secretion in a clinical setting, even though it is a measure of steady-state insulin secretion and should not be interpreted as a measure of the first phase.

Diabetes status could in theory influence the correlation between the reference and surrogate measure, but this is not supported by our data on type 2 diabetes and pre-diabetes as compared with that on NGT. Only a few studies reported stratified correlations for pre-diabetes. On the other hand, there is a low heterogeneity of correlation coefficients in pre-diabetes across the examined studies. In this meta-analysis it is shown that IGI(30), Stumvoll’s first-phase secretion and HOMA-beta all have consistent correlation coefficients to reference methods for pre-diabetes. It is especially important to accurately capture variation in insulin secretion for pre-diabetes because early dysfunction can be present without a profound change in glucose levels.^27^

The high heterogeneity in the majority of our meta-analyses can partially be explained by different methods of measuring and calculating insulin secretion in the reference methods. Insulin secretion expressed as AIR10min (insulin secretion during the first 10 min calculated as the AUC) was the most common reference. There are other possible reasons for the high heterogeneity in our analyses, including differences in age, sex, BMI, diabetes duration, fasting glucose, Hemoglobin A1c (HbA1c), treatment and technical differences. We could not stratify our analyses on these factors, since the included studies did not report correlations by these groups. There was also a large variation in intra-individual reproducibility among indices as regards measuring insulin secretion. Intra-individual variability is generally higher for insulin-based indices than for C-peptide-based measurements,^28^ which is also reflected by a high heterogeneity across surrogate measures of insulin resistance.^29^ All of the studies in our meta-analysis used the same insulin measurement assays for both surrogate and reference measurements, which argues against increased variation originating from a technical variation across different immunoassays.^30^ A further source of variation with insulin-derived measures could be a considerably shorter plasma half-life of insulin as compared with C-peptide combined with a physiologically pulsatile insulin secretion.^24^

This review of the correlation between reference and surrogate measures of first-phase insulin secretion is the largest to have been conducted; it includes comparative work and presents all available data. The screening of articles and data extraction was performed by two researchers separately to reduce inclusion bias.

One weakness of our meta-analyses is the small number of studies that compare surrogate and reference methods. While there was a larger number of studies available for HOMA-beta and IGI(30) (17 and 13, respectively), there were too few articles available for many other indices to conduct a meta-analysis. We decided not to include Embase database in our search since the database has a focus on pharmacovigilance; thus, there may have been validation studies that we did not include in our meta-analysis. In line with the Cochrane handbook we decided that the first step of the literature search was done by one author only.^12^ This is a possible limitation because of the risk of missing potential studies. There are other methodological limitations regarding the search strategy, the information about the breakdown for each database and the number of duplicates was not saved. This is of course an obstacle for verification of the method.

Another limitation is the different ways of calculating insulin secretion during its first phase. It would be useful for epidemiological studies to know which surrogate measure to use for different glycemic groups, for example, IGT and NGT. Because the first phase is blunted early in the natural history of type 2 diabetes it can be measured and used as a prognostic index, and it would therefore be interesting to restrict the analysis to participants with pre-diabetes. However, too few studies investigated subgroups with specific clinical characteristics to allow a conclusion to be drawn. The statistical analysis plan was not predefined in detail, which we consider in retrospect a limitation.

In this systematic review, validation studies that compare surrogate and reference measures of first-phase insulin secretion are summarized. The indices with the highest correlation to reference methods are Kadowaki and Stumvoll’s first-phase secretion. This shows that measurements of insulin and glucose during the first 30 min of an OGTT are sufficient for a reliable estimate of insulin secretion. But the correlations between surrogate measures and the reference examination for first-phase insulin secretion are modest at the most, which is explained by the reference method being an intravenous test. However, in epidemiological studies, only surrogate measures are affordable and may even present a more physiological measure. The most validated surrogate measures are HOMA-beta based on one fasting blood sample, and IGI(30) measured 30 min after an OGTT; the first being an estimate of basal insulin secretion and the latter a measure of early secretion after an oral glucose bolus.

## Supplementary material

10.1136/bmjdrc-2024-004256 online supplemental file 1

## Data Availability

Data are available upon reasonable request.
