# Supplementary material for: Surrogate measures of first-phase insulin secretion versus reference methods intravenous glucose tolerance test and hyperglycemic clamp: a systematic review and meta-analyses
Source: BMJ Open Diabetes Res Care. 2024 Jul 16;12(4):e004256. doi: 10.1136/bmjdrc-2024-004256 (PMC11268049; doi:10.1136/bmjdrc-2024-004256)
Supplement: online supplemental file 1 [file bmjdrc-12-4-s001.docx]

## Supplementary Table 1, Study characteristics

| **Study** | **Country** | **Number of Participants**  **(Proportion women, %)** | **Subject Category** | **Age**  Mean  (SD) | **BMI**  Mean  (SD) | **Surrogate meassure** | **Reference method** | **Method of Correlation** |
| --- | --- | --- | --- | --- | --- | --- | --- | --- |
| Azzi, 2019(1) | Belgium | 53 (66) | NGT | 36 (13,0) | 24,9 (5,4) | \| OGTT-IGI(30), \| \| --- \| \| HOMA- beta \| | IVGTT, Glucose (0.3 g/kg) was infused IV U/kg) and blood was sampled at times -15, -5 and -1 min before glucose administration, and at 2, 3, 4, 5, 6, 8, 10, 14, 16, 19, 22, 23, 24, 25, 27, 30, 35, 40, 50, 60, 70, 80, 90, 100, 120, 140, 160 and 180 min ^2^. Insulin secretion was quantified as the AIR_g_ over the first 10 minutes of the IVGTT. | Pearson |
| Basu, 2003(2) | USA | 88 (46) | NGT | --- | 26,0  (0,6) | Meal test, minimal model, Phi_dynamic_, Phi_static_, Phi_total*_ | IVGTT. Glucose (0,3g/kg) was given at time=0. Samples were drawn at -120,-30,-20,-10,-0,2,4,6,8,10,15,20 etc. Insulin was given at 20 minutes. Secretion was calculated with minimal model: Phi_1,_ Phi_2,_ Phi_total*_ | Pearson |
| Bonadonna, 2003(3) | Italy | 54 (78) | NGT, T2D | 47,5  (1,4) | 26,6  (0,7) | OGTT-Beta-index(4) | HGC-A bolus of glucose (20%) to raise and maintain glucose levels at 10mmol/l. Blood samples were collected -30-0, every 2,5 minutes between 0-10 minutes and every 5 minutes thereafter. 1st phase was calculated with minimal model. (3) | Spearman |
| Chang, 2006(5) | USA | 214 (61) | NGT, IGT | 56 (6,4) | 26 (4.1) | Log HOMA-beta | IVGTT-log-AIR,  calculated as the mean rise in plasma insulin above baseline at 3, 4, and 5 min after iv glucose administration  (50% dextrose, 0,3g/kg). | Pearson |
| Chiu, 2001(6) | USA | 105 (41) | NGT | 26 (1) | 24.79 (0.44) | OGTT-Stumvoll’s first- and second-phase secretion | HGC-1st phase  Participants received a bolus of 50% dextrose solution based on their body surface area (11.4 g/m2 ) at 0 min. Continuous infusion of 30% dextrose solution was commenced at 15 min were adjusted every 5 min based on the prevailing plasma glucose levels, to maintain a plasma glucose level around 10 mm toward 180 min The first-phase insulin response (1stIR) was the sum of plasma insulin levels during the first 10 min (2.5, 5, 7.5, and 10 min) | Pearson |
| Cobelli, 2007(7) | USA | 205 (43) | NGT | 46,2  (0,7) | 26 (0,5) | HOMA-beta | IVGTT-Minimal model described in Cobelli et al.(7) | Pearson |
| Coppack, 1991(8) | UK | 247 (---) | T2D | 53,1 (1,5) | 28,1 (0,5) | HOMA-beta | IVGTT-Glucose, 20g/m-2 body surface area, was given and samples were taken twice in fasting state and 13 times after injection. AIR, was calculated as incremental AUC 0-5 minutes after administration of glucose iv. | Spearman |
| Cretti, 2001(4) | Italy/Finland | 20 (---) | NGT, IGT, T2D | 64.2 (0.8) | 27.0 (0.8) | OGTT-Beta-index(4) | IVGTT. A bolus of 0,3g/kg and samples were drawn at 0,2,4,6,8,10 minutes. AIR was calculated as incremental AUC 0-10 minutes. | Pearson |
| Festa, 2008(9) | USA | 1380 (56) | NGT, IGT T2D | 57  (---) | 29,6  (---) | HOMA-beta | FSIGT, description could not be found. AIR was calculated as the mean concentration of insulin at 2 and 4 minutes | Spearman |
| Hammana, 2009(10) | Canada | 16 (44) | NGT | 24,8  (4,2) | 22.4  (1,6) | HOMA-beta, OGTT-IGI(30), Stumvolls first-phase secretion | IVGTT. A bolus of 20% glucose (0,3g/kg) was given and samples were drawn at -10,0,2,4,6,8,10,20,30,40,50,60. AUC was calculated for Insulin 0- 10 minutes. | Pearson |
| Hansen, 2007(11) | Denmark | 286 (56) | NGT, IGT | 51,5  (8,5) | 25,6  (3,7) | HOMA-beta, OGTT-Stumvolls first-phase secretion, BIGTT0-30-120, BIGTT0-60-120 | IVGTT. A bolus of glucose(0,3g/kg, 50%solution) was given and samples were taken at 2,3,4,5,6,8,10,12,14,16,19,22,23 etc. AIR was calculated as incremental insulin AUC 0-8 min. | Pearson |
| Hanson, 2000(12) | USA | 398 (---) | NGT, IGT | --- | --- | HOMA-beta, OGTT-IGI(30), 30 min insulin, CIR30, I30/G30, I30/I0, I120/G120, CIR120 | IVGTT, AIR, insulin concentration above basal level determined 3-5 minutes after bolus of 25 g iv glucose. | Pearson |
| Hermans, 1999(13) | UK | 24 (---) | NGT, IGT T2D | 58,7  (9,6) | 28,6  (4,7) | HOMA-beta | IVGTT- 0,3g/kg, (50%solution) glucose was given. Blood samples were drawn at -15,-10,-5,0,3,4,5,6,8,10,12,14,16 and 19. (At 20 min insulin infusion was given) (AIRg ) was calculated with the minimal model (version 2.0, MinMod) | Pearson |
| Lehtovirta, 2005(14) | Finland | 54 (34) | NGT | 64,8  (4,3) | 26,7  (0,6) | OGTT-Beta-index(4) | IVGTT-0,3g/kg wt of glucose was given and samples were drawn at 0,2,4,6,8,10,20,30,40,50,60 min. Readily releasable insulin (RRI; the first peaking phase of insulin secretion during IVGTT; pmol·l−1 was calculated in a modified minimal model. | Pearson |
| Maki, 2011(17) | USA | 24 (---) | NGT |  |  | HOMA-beta | IVGTT- 300 mg/kg wt bolus (50%solution) was given at time 0. Samples were collected at -10,-5,3, 5,7,10,12,14,16,19,22,25,30,40,50,60,75,90,120,150,180 min. AIR to intravenous glucose (AIRg) was defined as the incremental area under the insulin curve for the period 0 to 10 minutes. | Pearson |
| Mari, 2008(18) | Italy | 221 (87) | NGT, IGT T2D | 41,3  (9) | 28,9  (4,8) | OGTT-IGI(30) | IVGTT-0,3 g/kg wt was given. AIR, calculated as the mean insulin concentration increment above fasting value 0-8 min after bolus injection was used as a measure of first phase. | Not described. |
| Marini, 2012(19) | Italy | 305 (58) | NGT, IGT | 40  (9,3) | 30,3  (3,2) | \| OGTT-IGI(30), \| \| --- \| \| (AUC30Insulin/ AUC30Glucose ) \| \| Stumvoll first-phase secretion, \| \| HOMA-beta \| | IVGTT-Glucose (300mg/kg in a 50%solution) was given and samples were taken at 2,4,6,8,10,20,30,40,50,60 min. AIR, calculated as incremental AUC for insulin during the first 0-10 min using the trapezoidal rule. | Pearson |
| Mohandas 2018(21) | UK | 33 (0) | NGT | 54,8  (7,4) | 29,8  (2,7) | Meal Test-First phase | HGC-First Phase, calculated as incremental AUC 0-10 min after administration of glucose infusion to maintain glucose level at 6.9 mmol/l over fasting level. | Pearson |
| Nijpels, 1994(22) | The Netherlands | 16 (---) | NGT, IGT T2D | ---Art | --- | \| OGTT-Insulin30/Glucose30 \| \| --- \| \| AUC insulin/glucose \|   Insulin 120 (log) | HGC-After administration of a 150mg/kg 20% glucose solution glucose levels were maintained at 10mmol/l. First phase: delta AUC  0-10 min, second phase: mean insulin level over the last 20 minutes(160-180 min) | Pearson |
| Okuno, 2013(23) | Japan | 101 (47) | NGT, IGT T2D | 42,1  (11,7) | 24,9  (5,0) | OGTT-(120) CPI | HGC- An infusion of glucose (9622mg/m^2^) followed by a variable dose of glucose to maintain a concentration of 200 mg/dl for 90 minutes. First Phase was defined as the incremental AUC for insulin from 0-10 minutes. | Pearson |
| Overgaard, 2006(24) | Denmark | 40 (40) | NGT | 29,2  (1,7) | 25,1  (1,0) | OGTT, A0, gamma | IVGTT. An infusion of a 25% solution of glucose (300mg/kg wt, max 25 g) was given. Samples were collected at -30,-20,-10,-1,2,3,4,5,6,8,10,12,14,16,19,22,25,30,40,50,60,70,80,90,100,110,120,140,160,180 min. A0 was calculated as first phase measure. (24) | Pearson |
| Philips, 1994(25) | UK | 108(46) | NGT, IGT | 52,0  (---) | 26,9  (---) | HOMA-beta  OGTT-IGI(30), Insulin0/Glucose0 | IVGTT-Glucose (50%soultion 0,3g/kg wt) was administered. The 3 min secretion was used as a measure of first phase secretion. | Pearson |
| Santos, 2015(26) | Chile | 57 (100) | NGT | 27.2 (6.3) | 23.4 (2.9) | OGTT-AUCInsulin/AUCGlucose | IVGTT-An I.v. dose of 0,3 g glucose /kg wt as 50% solution. Measurements were taken at -15,-5, 2,3,4,5,6,8,10 min to calculate AIR. | Spearman |
| Shankar, 2016(28) | USA | 62 (53) | NGT, IGT T2D | 47,1  (8,8) | 33,1  (3,5) | Meal tolerance test, total 10 min | IVGTT-Following baseline sampling (-30,-15 and 0 min) a 300 mg/kg wt glucose bolus was administered with sampling at 2, 4, 8, 19 minutes. AIR, calculated as incremental AUC above average basal level 0-10 min. | Spearman |
| Stancakova, 2009(29) | Finland | 287 (0) | --- | --- | --- | HOMA-beta  OGTT-IGI(30), Stumvoll’s first- and second-phase secretion, AUC30insulin/AUC30glucose,  AUC120Insulin/AUC120Glucose,  Fasting insulin, Insulin120,  AUCinsulin0-120 | IVGTT- A bolus of glucose (300 mg/kg in a 50% [wt/vol.] solution) was given within 30 s. Blood samples for the measurement of plasma glucose and insulin were drawn at –5, 0, 2, 4, 6, 8, 10, 20, 30, 40, 50 and 60 min. AIR was calculated as the AUC the first 0-10 minutes after glucose bolus. | Spearman |
| Steil, 2004(30) | USA | 17(18) | NGT | 50 (2) | 25.0 (0.7) | Mealtest, minimal model | HGC-Glucose (50%solution, 0,15g/kg) was given at time 0 and a glucose infusion (20%) was started to maintain a glucose level 10 mmol/l for 180 minutes. First phase was calculated as incremental AUC 0-10 min, samples taken at -15,-10,-1, 2,3,4,5,6,8,10 minutes. | Pearson |
| Stumvoll, 2k000(31) | Germany | 28 (---) | T2D | 54 (2) | 27,7 (0,9) | HOMA-beta,  OGTT-AUCinsulin/AUCGlucose,  Insulin120, (Insulin30 - Insulin0)/Glucose30) | HGC-Glucose levels were raised to 10 mmol/l for 180 minutes. First phase was calculated as the sum of plasma insulin concentrations at 2.5, 5, 7.5, and 10 min of the  hyperglycemic clamp experiment minus the  mean basal plasma insulin concentration | Regression analysis |
| Stumvoll, 2000(32) | Germany | 104 (62,5) | NGT | 45 (10) | 27.6 (0.5) | OGTT-IGI(30), Stumvoll’s first- and second-phase secretion, Insulin30-0/Glucose30, Insulin30-0  HOMA-beta | HGC-Glucose levels were raised to 10 mmol/l for 180 minutes. First phase was calculated as the sum of plasma insulin concentrations at 2.5, 5, 7.5, and 10 min of the  hyperglycemic clamp experiment minus the  mean basal plasma insulin concentration | Spearman |
| Taniguchi, 2000(33) | Japan | 25 (---) | T2D | 45.4 (2.3) | 20.2 (0.6) | OGTT-IGI(30), Insulin30-0/Glucose30, Insulin30-0, HOMA-beta | IVGTT-Glucose(300mg/kg body wt) was administered i.v. Insulin secretion was expressed as the area under the insulin curve between 0 and 10 min after an intravenous glucose injection, using the trapezoidal method. | Pearson |
| Tripathy, 2004(34) | Sweden/Finland | 218 (---) | NGT, IGT T2D | --- | ---- | HOMA-beta  OGTT-IGI(30) | IVGTT-0,3 g glucose/kg body wt of a 50% glucose solution was given at time 0. Blood samples were taken at time: -10,0,2,4,6,8,10, 20,40,50,60,120 min. AIR was calculated as the incremental AUC 0-10 min using the trapezoidal method. | Pearson |
| Tura, 2006(35) | Italy | 145 (---) | NGT, IGT T2D, former gestational diabetes | --- | --- | OGTT, IGI(30) | IVGTT-AIR was calculated as mean incremental concentration in the 3-5 minutes following glucose bolus. AIR was then divided by the incremental glucose peak to obtain the same unit as IGI(30). | Regression analysis |
| Van Haeften, 1998(36) | Holland | 42 (---) | NGT | 45,3 (1,4) | 26,3 (0,8) | OGTT-Log Insulin30, Insulin45 | HGC-A HGC was performed during 180 minutes aiming at a glucose level of 10mmol/l, starting with an iv bolus of 35mg glucose/kg per mmol/l intended glucose increase. Log transformed plasma insulin levels for the first 10 minutes were used as first phase. Surrogate measures correlated with 4minutes plasma insulin levels. | Pearson |
| Wagner, 2020 | Germany | 309-IVGTT  76-HGC | NGT, IGT T2D |  |  |  | IVGTT, AIR, calculated as incremental AUC 0-10 min.  HGC-calculated at steady state. | Pearson |

Supplementary table 2. Quality analysis of studies included in main analysis.

| **Study** | **RISK OF BIAS** | | | |  | **APPLICABILITY CONCERNS** | | | **RISK OF BIAS SUMMARY** |
| --- | --- | --- | --- | --- | --- | --- | --- | --- | --- |
|  | **PATIENT SELECTION** | **INDEX TEST** | **REFERENCE STANDARD** | **FLOW AND TIMING** | **ANALYTICAL TECHNIQUE** | **PATIENT SELECTION** | **INDEX TEST** | **REFERENCE STANDARD** |  |
| Azzi 2019 | ☺ | ☺ | ☺ | ☺ | ☺ | ☹ | ☺ | ☺ | Unclear |
| Basu 2003 | ☺ | ☺ | ☺ | ☺ | ☺ | ☺ | ☺ | ☺ | Low |
| Bonadonna, 2003 | ☺ | ☺ | ☺ | ☺ | ☺ | ☺ | ☺ | ☺ | Low |
| Chang, 2006 | ☺ | ☺ | ☺ | ☺ | ☺ | ☺ | ☺ | ☺ | Low |
| Chiu, 2001 | ☺ | ☺ | ☺ | ☺ | ☺ | ☺ | ☺ | ☺ | Low |
| Cobelli, 2007 | ☺ | ☺ | ☺ | ☺ | ☺ | ☺ | ☺ | ☺ | Low |
| Coppack, 1991 | ☺ | ☺ | ☺ | ? | ☺ | ☺ | ☺ | ☺ | Unclear |
| Cretti, 2001 | ☺ | ☺ | ☺ | ? | ☺ | ☺ | ☺ | ☺ | Unclear |
| Festa, 2008 | ☺ | ☺ | ☺ | ☺ | ☺ | ☺ | ☺ | ☺ | Low |
| Hammana, 2009 | ☺ | ☺ | ☺ | ☺ | ☺ | ☹ | ☺ | ☺ | Unclear |
| Hansen, 2007 | ☺ | ☺ | ☺ | ☺ | ☺ | ☺ | ☺ | ☺ | Low |
| Hanson, 2000 | ☺ | ☺ | ☺ | ☺ | ☺ | ☺ | ☺ | ☺ | Low |
| Hermans, 1999 | ☺ | ☺ | ☺ | ? | ☺ | ☺ | ☺ | ☺ | Unclear |
| Lehtovirta, 2005 | ☺ | ☺ | ☺ | ☺ | ☺ | ☺ | ☺ | ☺ | Low |
| Maki, 2011 | ☺ | ☺ | ☺ | ☺ | ☺ | ☺ | ☺ | ☺ | Low |
| Mari, 2008 | ☺ | ☺ | ☺ | ☺ | ☺ | ☺ | ☺ | ☺ | Low |
| Marini, 2012 | ☺ | ☺ | ☺ | ☺ | ☺ | ☺ | ☺ | ☺ | Low |
| Mohandas, 2018 | ☺ | ☺ | ☺ | ☺ | ☺ | ☺ | ☺ | ☺ | Low |
| Nijpels, 1994 | ☺ | ☺ | ☺ | ☺ | ☺ | ☺ | ☺ | ☺ | Low |
| Okuno, 2013 | ☺ | ☺ | ☺ | ☺ | ☺ | ☺ | ☺ | ☺ | Low |
| Overgaard, 2006 | ☺ | ☺ | ☺ | ☺ | ☺ | ☺ | ☺ | ☹ | Unclear |
| Philips, 1994 | ☺ | ☺ | ☺ | ☺ | ☺ | ☺ | ☺ | ☺ | Low |
| Prystupa, 2022 | ☺ | ☺ | ☺ | ☺ | ☺ | ☺ | ☺ | ☺ | Low |
| Santos, 2015 | ☺ | ☺ | ☺ | ☺ | ☺ | ☺ | ☺ | ☺ | Low |
| Shankar, 2016 | ☺ | ☺ | ☺ | ☺ | ☺ | ☺ | ☺ | ☺ | Low |
| Stancakova, 2009 | ☺ | ☺ | ☺ | ? | ☺ | ☺ | ☺ | ☺ | Unclear |
| Steil, 2004 | ☺ | ☺ | ☺ | ☺ | ☺ | ☺ | ☺ | ☺ | Low |
| Stumvoll, 2000 | ☺ | ☺ | ☺ | ☺ | ☺ | ☺ | ☺ | ☺ | Low |
| Stumvoll, 2000 | ☺ | ☺ | ☺ | ☺ | ☺ | ☺ | ☺ | ☺ | Low |
| Taniguchi, 2000 | ☺ | ☺ | ☺ | ? | ☺ | ☺ | ☺ | ☺ | Unclear |
| Tripathy, 2004 | ☺ | ☺ | ☺ | ? | ☺ | ☺ | ☺ | ☺ | Unclear |
| Tura, 2006 | ☺ | ☺ | ☺ | ☺ | ☺ | ☺ | ☺ | ☺ | Low |
| Van Haeften, 1998 | ☺ | ☺ | ☺ | ? | ☺ | ☺ | ☺ | ☺ | Unclear |
|  |  |  |  |  |  |  |  |  |  |

☺= low risk of bias. ☹=high risk of bias. ?=unclear risk of bias. For the RISK OF BIAS SUMMARY, a study is judged as unclear risk of bias if one but not more than one of the bias categories are assessed as high or unclear risk of bias.

Supplementary Table 3. Surrogate Indices with less than 3 validation studies, here presented with first author of study, number of participants and a correlation coefficient to reference method, hyperglycemic clamp, HGC, or intravenous glucose tolerance test, IVGTT.

| **Name** | **Subgroup** | **Number:** | **Surrogate index** | **Comparison** | **Correlation** | **P** |
| --- | --- | --- | --- | --- | --- | --- |
| Azzi | Total | 119 | C-pep(30-0)/Glucose(30-0) | IVGTT, AUC 0-10 min | \| 0.72 \| \| --- \| | <0.001 |
| Basu | \| NGT \| \| --- \| | 88 | Minimal model from meal tolerance test: **Phi-dynamic** | Minimal model from IVGTT: Phi1 | 0,45 | <0.001 |
| Chang | NGT | 214 | logHOMA-beta | Log AIR | 0.37 | <0.001 |
| Coppack | T2d | 247 | HOMA-beta | Absolut first phase | 0,73 | <0.001 |
| Coppack | T2D | 247 | HOMA-beta | Minimal model first phase | 0,67 | <0.001 |
| Coppack | T2D | 247 | HOMA-beta | Minimal model second phase | 0,49 | <0.001 |
| Hammana | Control | 16 | HOMA-beta | IVGTT0-4 min | 0,64 | <0,05 |
| Hammana | Control | 16 | HOMA-beta | IVGTTAIR0,4,6 min | 0,64 | <0,05 |
| Hammana | Control | 16 | HOMA-beta | IVGTT0-4-6 min | 0,68 | <0,05 |
| Hammana | Control | 16 | IGI(30) | IVGTT0-4 min | 0,49 | <0,05 |
| Hammana | Control | 16 | IGI(30) | IVGTTAIR0,4,6 min | 0,59 | <0,05 |
| Hammana | Control | 16 | IGI(30) | IVGTT0-4-6 min | 0,52 | <0,05 |
| Hammana | Control | 16 | Stumvoll’s first-phase secretion | IVGTT0-4 min | 0,62 | <0,05 |
| Hammana | Control | 16 | Stumvoll’s first-phase secretion | IVGTTAIR0,4,6 min | 0,72 | <0,05 |
| Hammana | Control | 16 | Stumvoll’s first-phase secretion | IVGTT0-4-6 min | 0,67 | <0,05 |
| Hansen | NGT | 258 | BIGTT-full | IVGTT, incremental AUC 0-8 minutes | 0,45 | <0,05 |
| Hanson | NGT+IGT | 398 | Insulin0 | IVGTT, AIR determined 3-5 minutes after bolus | 0,11 | <0,05 |
| Hanson | NGT+IGT | 398 | IGI120 | IVGTT, AIR determined 3-5 minutes after bolus | 0,27 | <0.001 |
| Hermans | Mixed | 24 | 2h CIGMA | IVGTT-AIR | 0,63 |  |
| Mohanda | Mixed | 33 | Meal tolerance test, first phase | HGC-first phase, calculated as the incremental AUC 0-10 minutes | 0,596 | <0,01 |
| Okuno | Mixed | 101 | OGTT (120)CPI(23) | HGC-AUCInsulin0-10min/Glucose10min | 0,608 | P<0,0001 |
| Okuno | Mixed | 101 | OGTT (120)CPI | HGC-AUCInsulin0-90min/Glucose90min | 0,777 | P<0,0001 |
| Okuno | Mixed | 101 | Fasting CPI (100xserum C-peptide level/glucose level) | HGC-AUCInsulin0-10min/Glucose10min | 0,649 | P<0,0001 |
| Okuno | Mixed | 101 | Fasting CPI (100xserum C-peptide level/glucose level) | HGC-AUC0-90min/Glucose90min | 0,721 | P<0,0001 |
| Okuno | Mixed | 101 | Postprandial CPI | HGC-AUC0-10min/Glucose10min | 0,588 | P<0,0001 |
| Overgaard | NGT | 40 | OGTT, A0 | IVGTT | 0,96 |  |
| Santos | NGT | 57 | OGTT: AUCInsulin/Glucose | AIR/glucose | 0,385 | 0,04 |
| Stancakova | NGT | 287 | DeltainsAUC120/DeltaGlucoseAUC120 | AUC0-10 min IVGTT | 0,375 | <0,001 |
| Tura | Mixed | 145 | OGTT-AUCI/AUC-glucose | Delta AIR insulin/delta glucose | 0,53 | <0,0001 |
| Tura | Mixed | 145 | OGTTAUC-CP/AUCglucose | Delta AIR Insulin/delta glucose | 0,77 | <0,0001 |
| Tura | Mixed | 145 | OGTTAUC-CP/AUCglucose | Delta AIR c-peptide/delta glucose | 0,56 | <0,0001 |
| Van Haeften | T2D-relatives | 21 | Log insulin 30 min | HGC-first phase 4 min | 0,555 | 0,0085 |
| Van Haeften | T2D-relatives | 21 | Log insulin 45 minutes | HGC-first phase 4 min | 0,66 | 0,0013 |
| Van Haeften | T2D-relatives | 21 | Log insulin 60 min | HGC-first phase 4 min | 0,542 | 0,011 |
| Van Haeften | NGT | 21 | Log insulin 30 minutes | HGC-first phase 4 min | 0,437 | 0,045 |
| Van Haeften | NGT | 21 | Log insulin 45 minutes | HGC-first phase 4 min | 0,453 | 0,037 |

**Abbreviations: OGTT;** oral glucose tolerance test; **NGT-**normal glucose tolerance; **IGT**-impaired glucose tolerance; **T2D**-type 2 diabetes; **HGC**-hyperglycemic clamp; **IVGTT**-intravenous glucose tolerance test; **AUC**-area under the curve; **AIR**- acute insulin response; **CFRD**; cystic fibrosis related diabetes

Supplementary Figure 1, PRISMA 2020 Flow diagram for systematic reviews

**Identification of studies via databases and registers**

Dublicates removed before screening (n = 952)

Records identified from:

PubMed (n=5 846)

Web of Science (n=5 677)

Cochrane Central (n=1 032)

**Identification**

Titles screened

(n =10 647)

Titles excluded

(n =10 309)

Articles for abstract review

(n =338)

Articles excluded

(n =194)

**Screening**

Articles selected for full reading

(n = 144)

Reports excluded:

No correlation coefficent (n=78)

Not adults (n=2)

No surrogate measure (n=2)

Not gold standard measurement of first phase: (n=43)

Articles included after contact with authors and retrieval of correlation coeffient. (n=7)

Studies included in review

(n =33 )

**Included**

Articles included through cross reference

(n = 7)

*From:*  Page MJ, McKenzie JE, Bossuyt PM, Boutron I, Hoffmann TC, Mulrow CD, et al. The PRISMA 2020 statement: an updated guideline for reporting systematic reviews. BMJ 2021;372:n71. Doi: 10.1136/bmj.n71

Supplementary Figure 2. Meta-analyses of correlations coefficients between surrogate measures of insulin secretion and reference methods, validated in less than three studies. The heterogeneity for each meta-analysis is specified below the figure for each surrogate measure.

1. CIR30
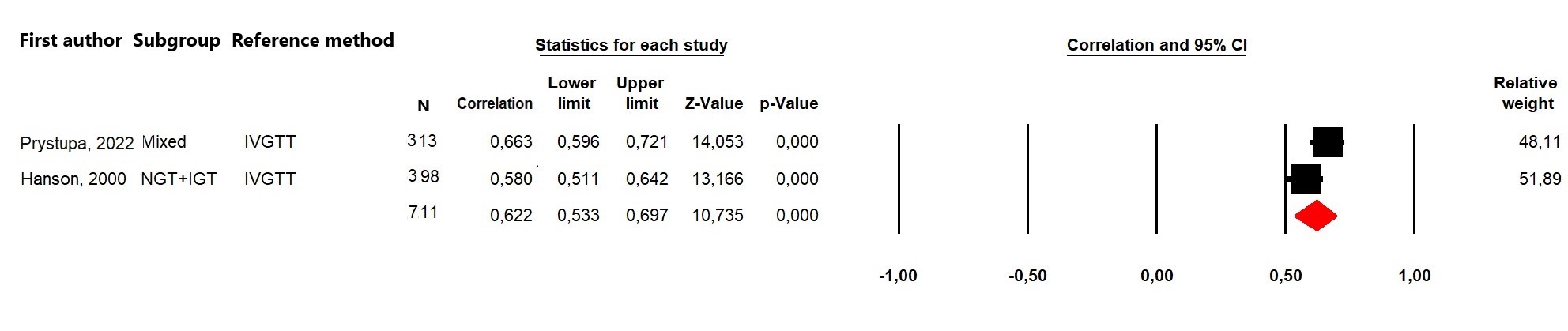


Heterogeneity: I^2^=69%

1. CIR120
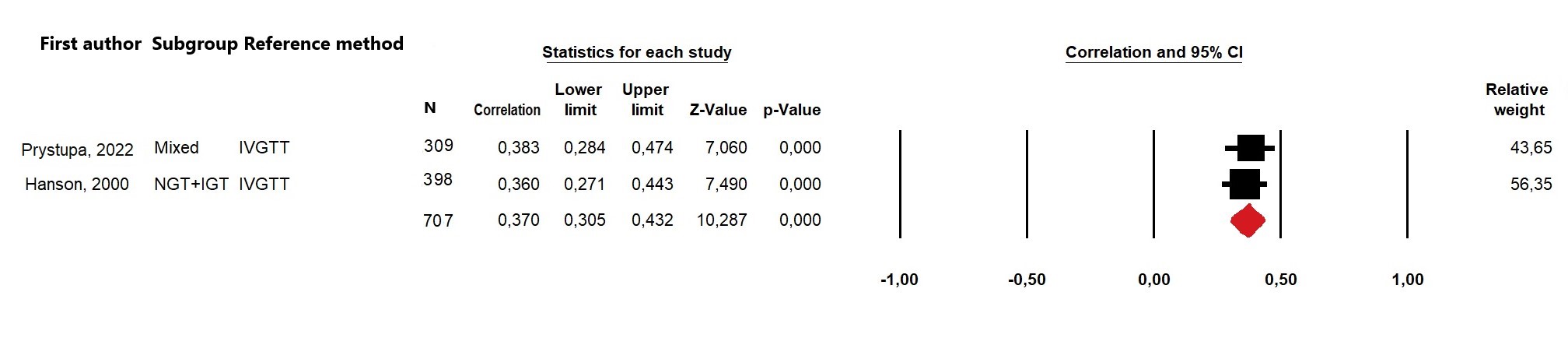


Heterogeneity: I^2^=0%

C) Insulin30-Insulin0
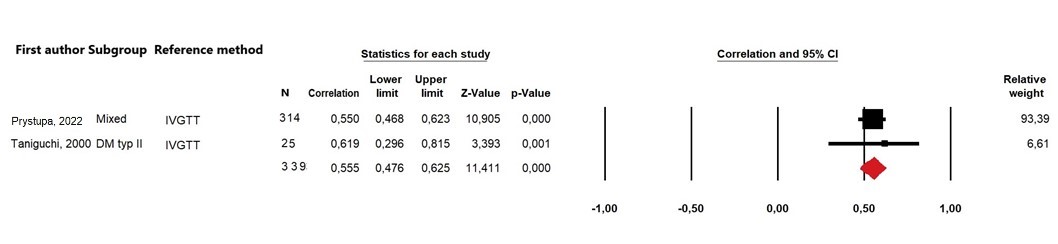


Heterogeneity: I^2^=0%

E) Insulin120/Glucose120
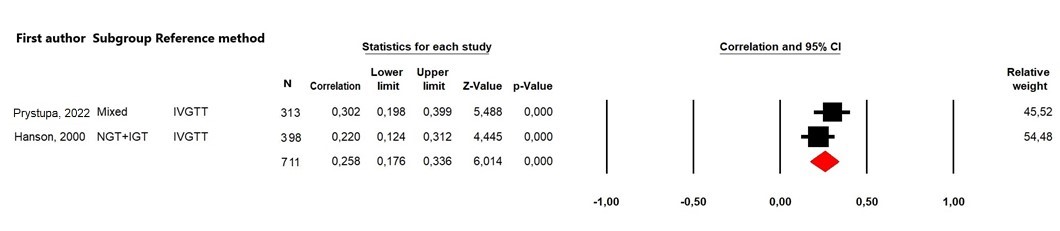


Heterogeneity: I^2^=26%

Supplementary Figure 3. Meta-analyses of correlations coefficients between surrogate measures and reference methods, stratified by glycemic status.

A) Correlation between IGI(30) and reference method for NGT, prediabetes and T2D.
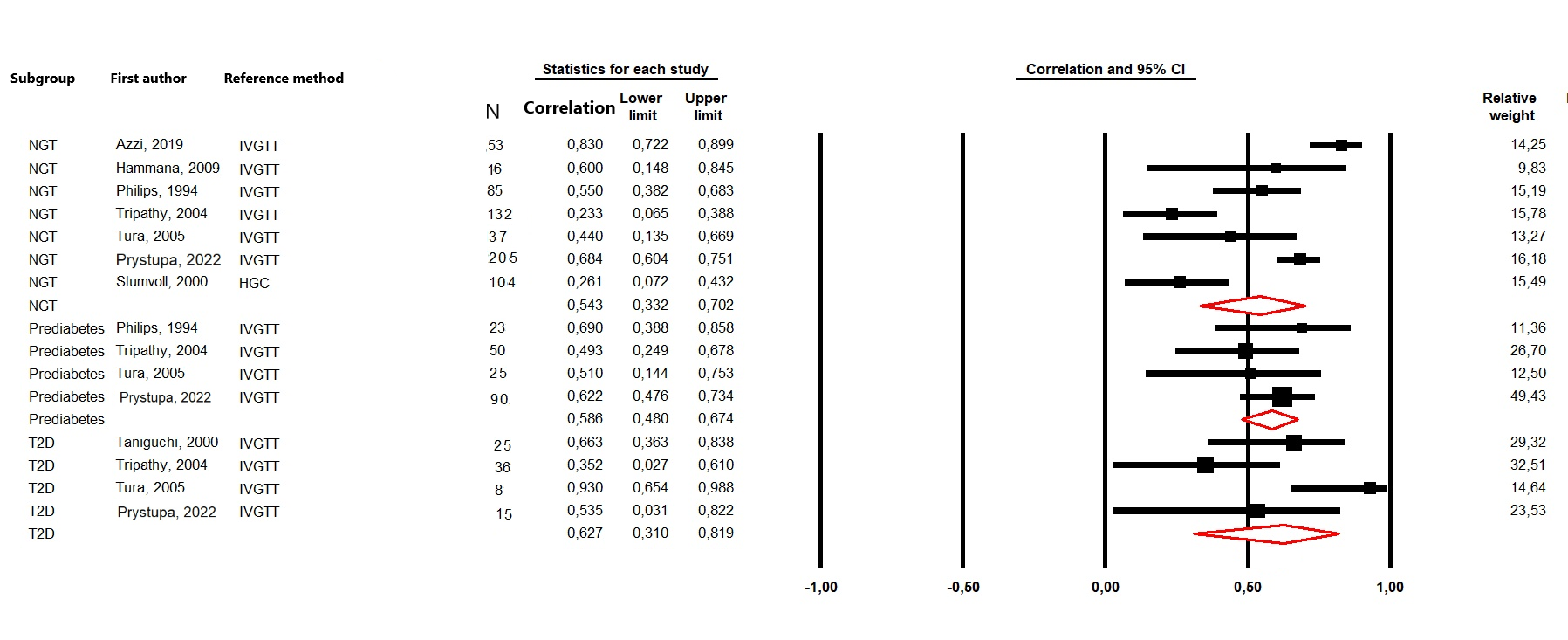


Heterogeneity: I^2^(NGT)=90%, p<0.05; I^2^(Prediabetes)=0%, p=0,58; I^2^(T2D)=63%, p<0.05; I^2^(overall)=80%, p<0.05

Difference between: p=0.862

1. Correlation between HOMA-beta and reference method for NGT, prediabetes and T2D.
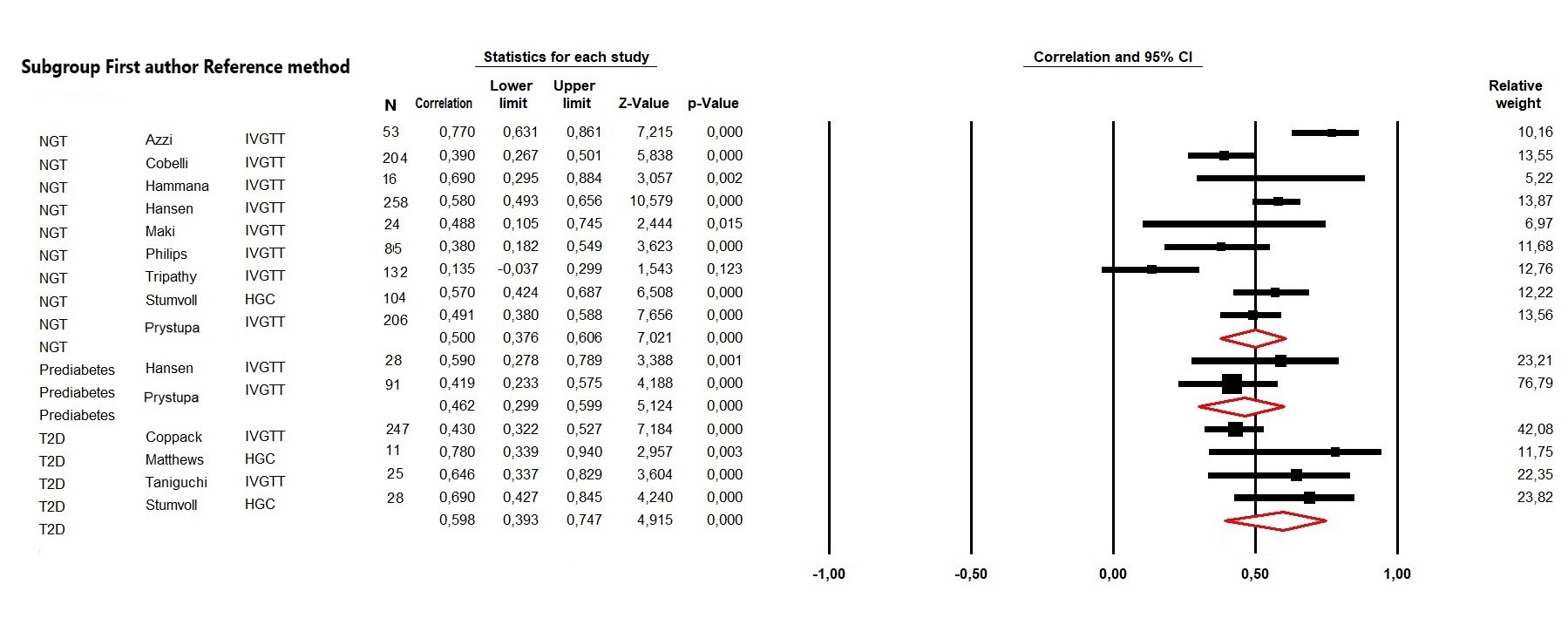


Heterogeneity: I^2^(NGT)=82%, p<0.05; I^2^(Prediabetes)=4%, p=0,31; I^2^(T2D)=58%, p=0.07; I^2^(overall)=73%, p<0.05

Difference between: p=0.536

1. Correlation between Stumvoll’s first phase secretion and reference method for NGT and prediabetes.
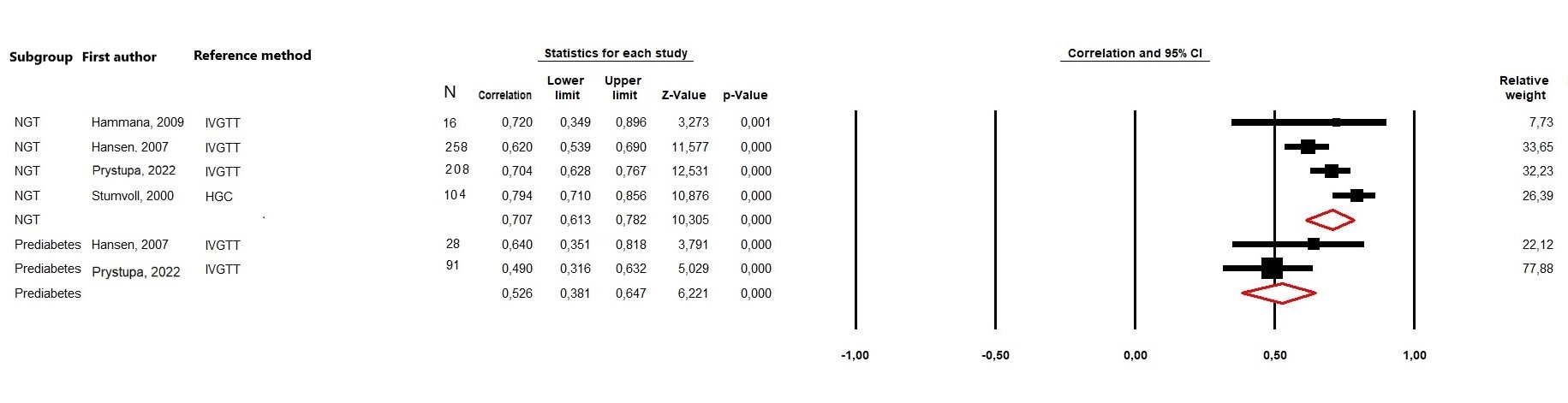


Heterogeneity: I^2^(NGT)=69%, p<0.05; I^2^(Prediabetes)=0%, p=0,33; I^2^(overall)=71%, p<0.05

Difference between: p<0.05

Supplementary Figure 4. Meta-analyses of correlation coefficients between surrogate measures and different measurements of first phase insulin secretion from the reference tests. The methods are described in Table 1 for each study. Heterogeneity and statistical difference between the methods is shown below the figure for each method.

1. Different first phase calculations in correlation with IGI(30). Calculation based on 1)Insulin levels above basal level 3-5 minutes after bolus; 2) 3min secretion; 3) AIR, the incremental area under the curve, for the first 8 minutes after bolus; 4) AIR for the first 10 minutes after bolus and 5) Delta AIR for the first 10 minutes; 6) The sum of plasma insulin concentrations at 2.5, 5, 7.5, and 10 min of the HGC minus the mean basal plasma insulin concentration.
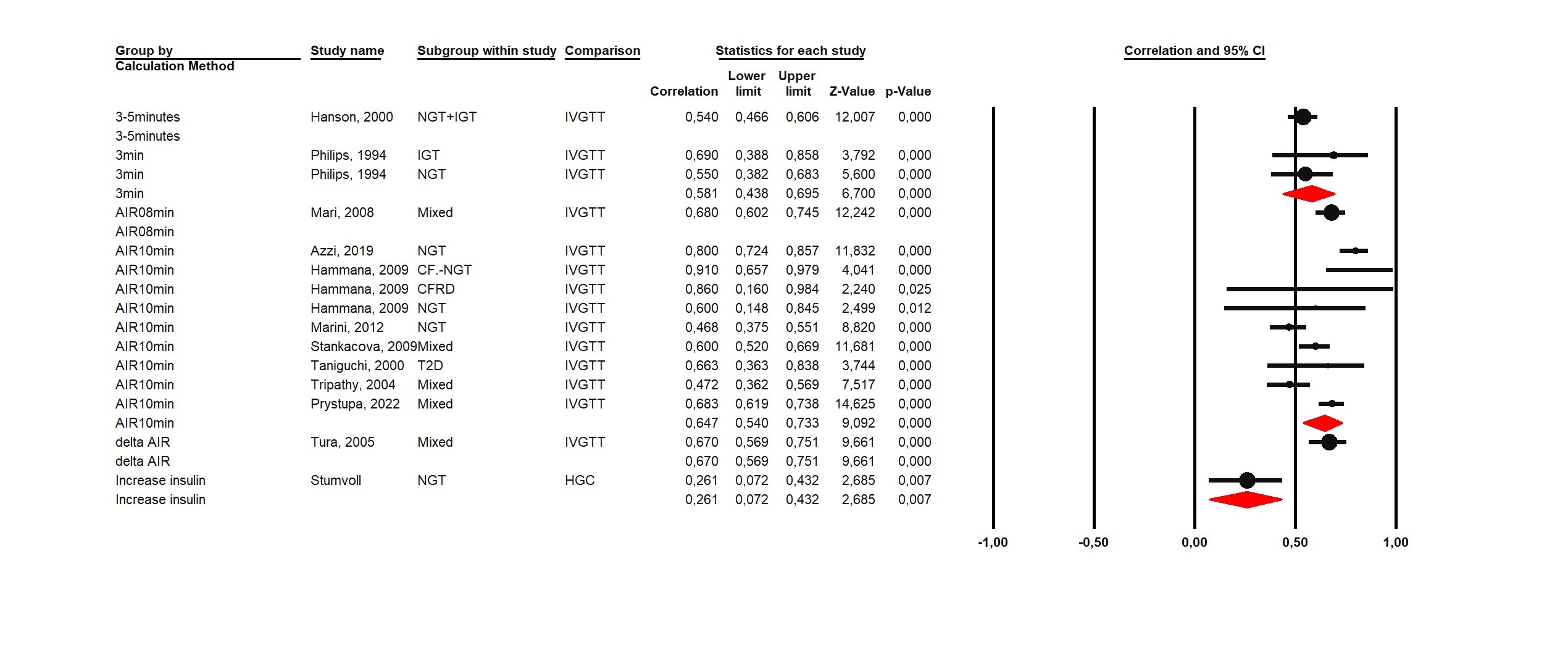


Heterogeneity: I^2^(3min)=0%; I^2^(AIR10min)=83%, p<0.05; I^2^(overall)=81%, p<0.05. Difference between: p<0.05

B) Different calculation of first face in correlation to HOMA-beta. HOMA-beta correlated to IVGTT calculations based on 1) Insulin levels 10-30 minutes into the IVGTT; 2) 3min secretion; 3) AIR, the incremental area under the curve, for 2-4 minutes; 4) AIR for the first 3-5 minutes; 5) AIR for the first 5 minutes; 6) AIR for the first 8 minutes; 7) AIR for the first 10 minutes and 8) the first phase calculated with minimal model.
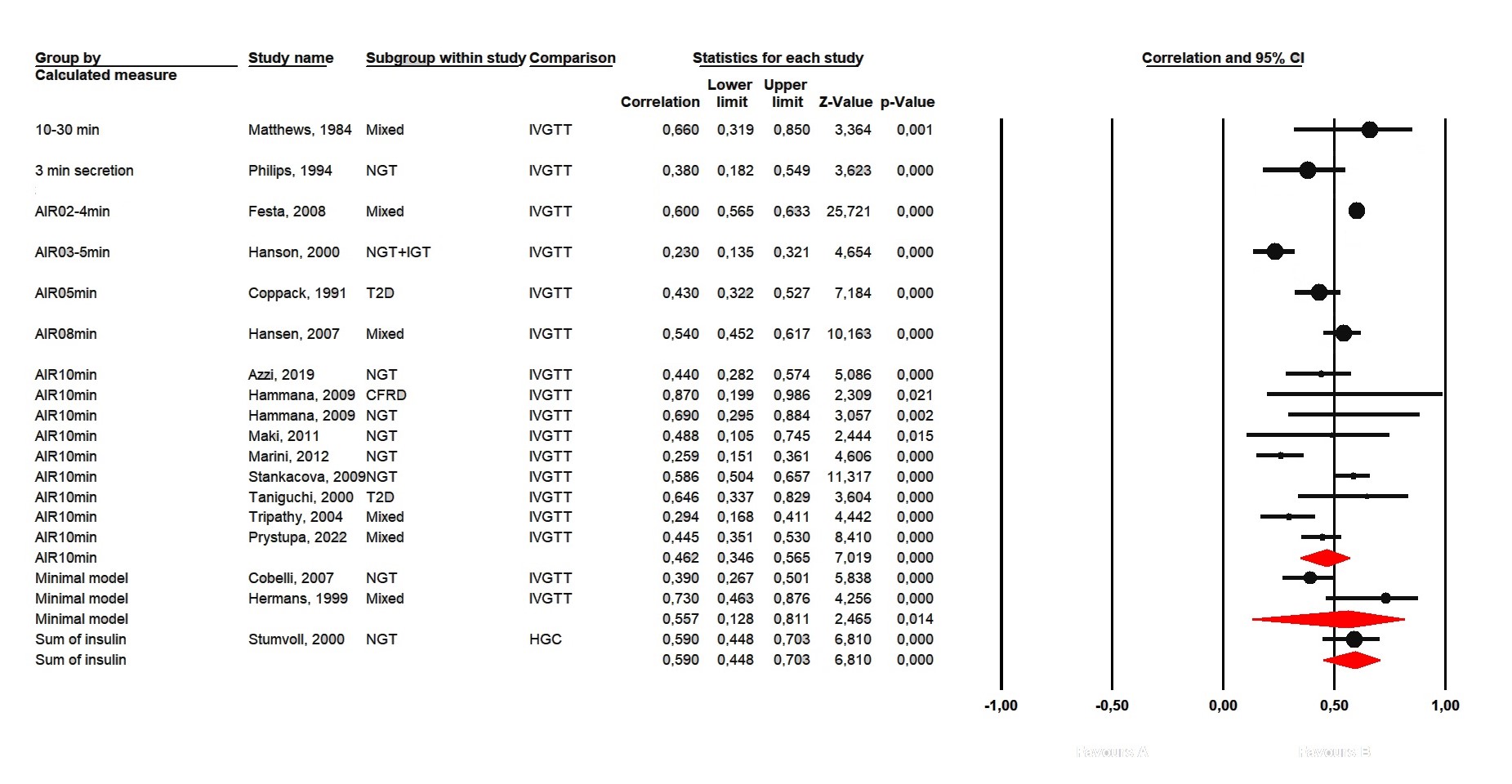


Heterogeneity: I^2^(AIR10min )=86%, p<0.05; I^2^(Min)=80%, p<0.05; I^2^(overall)=88%, p<0.05. Difference between: p<0.05

Supplementary Figure 5. Meta-analysis of correlation between IGI(30) and HOMA-beta to reference separated into groups with missing or complete participant data. Heterogeneity and statistical difference between groups are presented below the graphs.

1. HOMA-beta
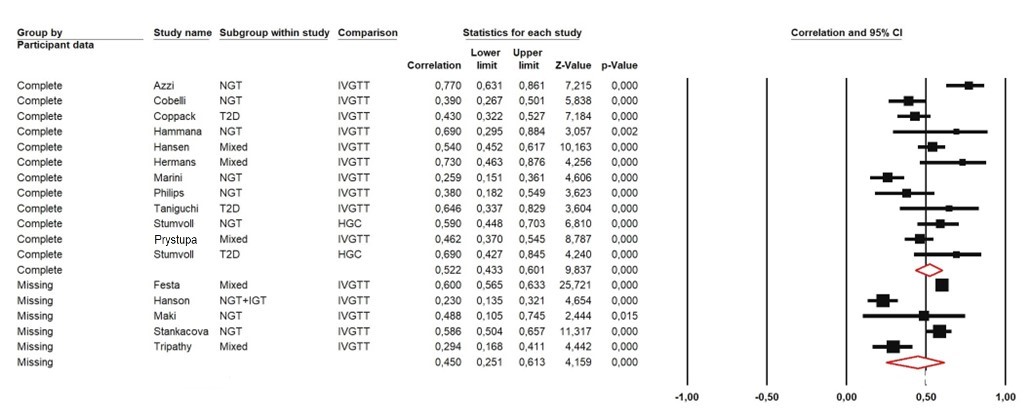


Heterogeneity: I^2^(complete)=78%, p<0.05; I^2^(missing)=95%, p<0.05; I^2^(overall)=88%, p<0.05. Difference between: p=0.468

1. IGI(30)
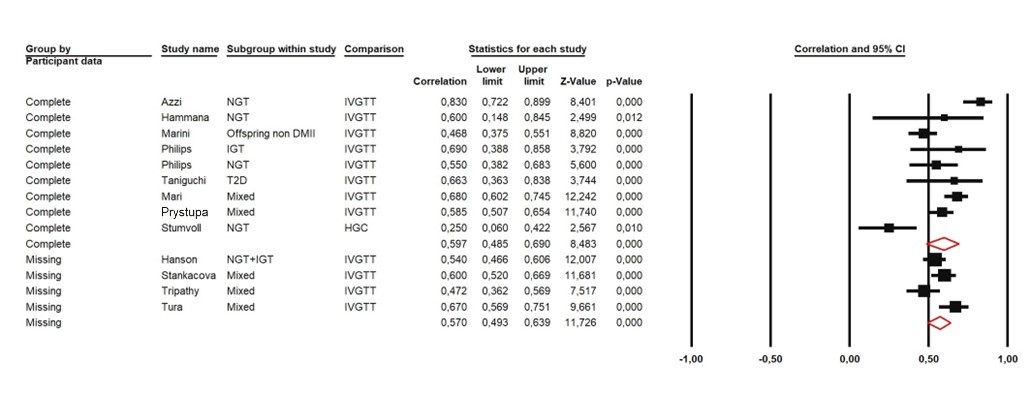


Heterogeneity: I^2^(complete)=82%, p<0.05; I^2^(missing)=66%, p<0.05; I^2^(overall)=78%, p<0.05. Difference between: p=0.676

Supplementary figure 6. Comparison of studies classified as low vs unclear risk according to Quaddas 2.

A: IGI(30)


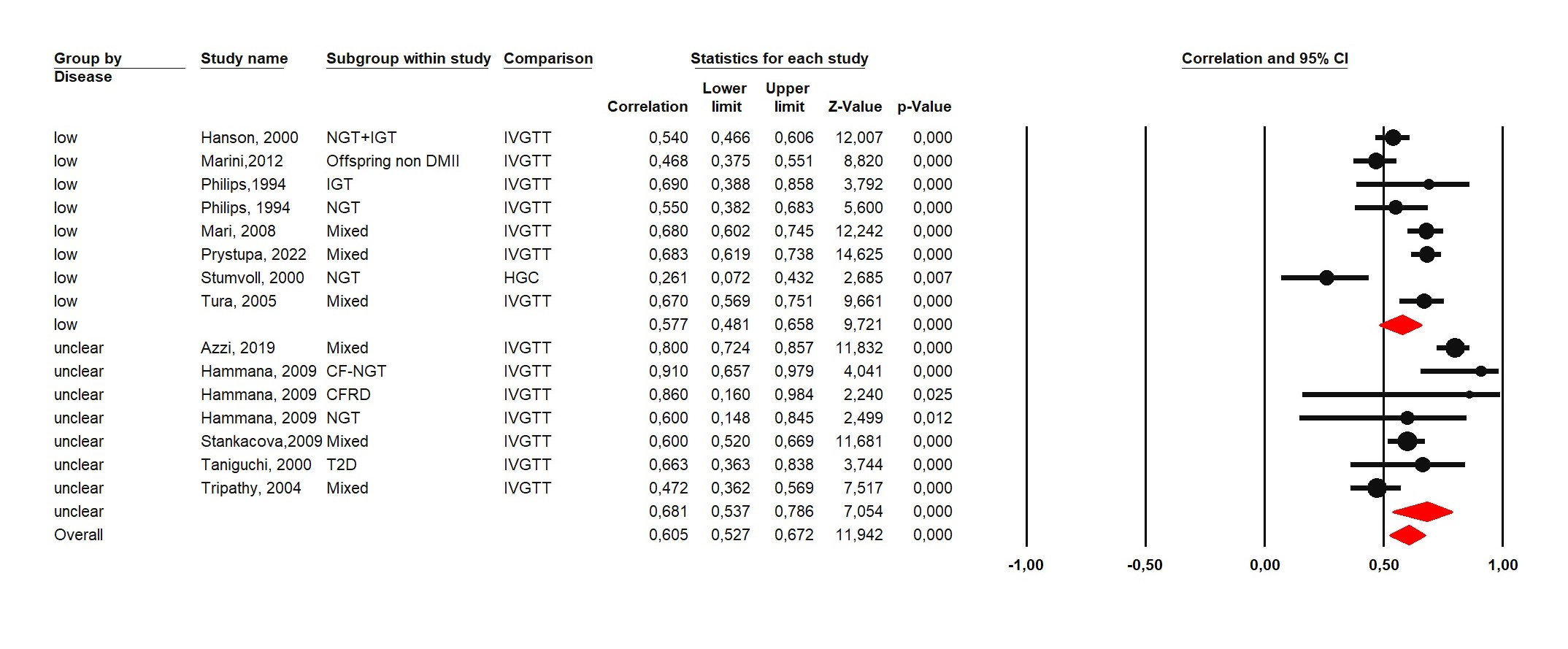


Heterogeneity: I^2^(low)=84%, p<0.05; I^2^(unclear)=81%, p<0.05; I^2^(overall)=82%, p<0.05. Difference between: p=0.201

B: HOMA-beta
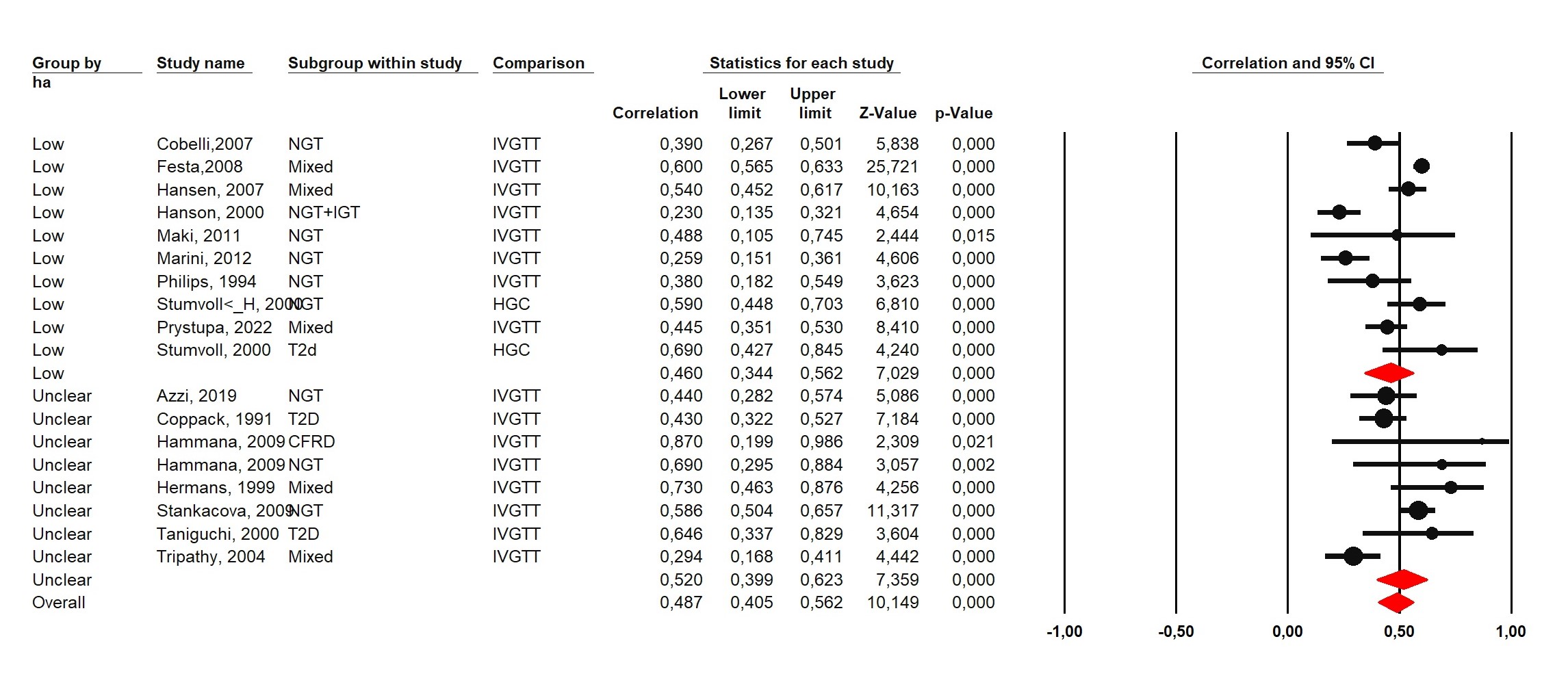


Heterogeneity: I^2^(low)=91%, p<0.05; I^2^(unclear)=73%, p<0.05; I^2^(overall)=87%, p<0.05. Difference between: p=0.454

Supplementary Figure 7. Random effect meta-analyses in comparison with fixed effect analyses.


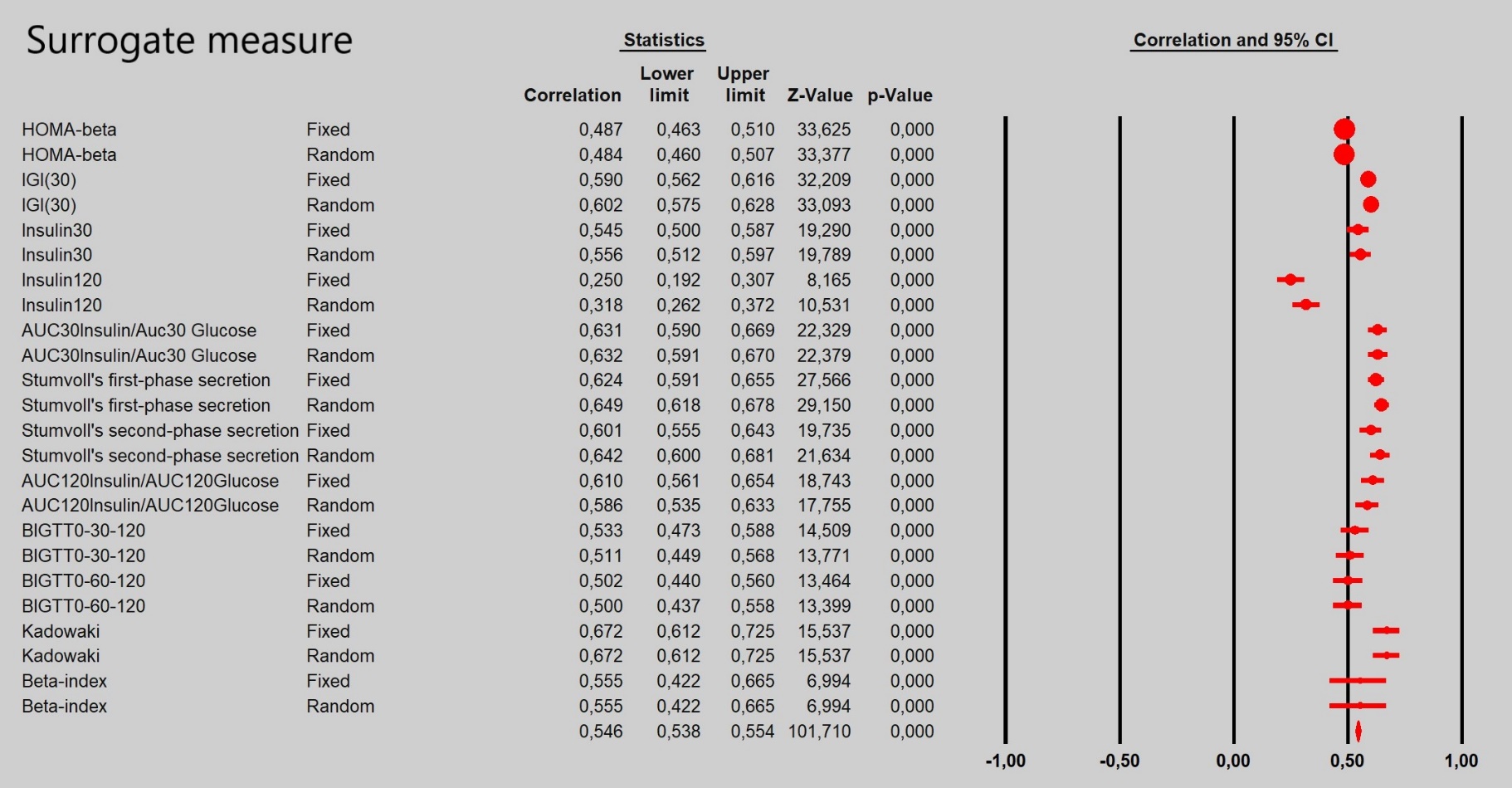


Supplementary Figure 8. Funnel plots of meta-analyses between the reference methods and surrogate measures where 3 or more studies with a correlation coefficient to the reference method were included .

1. HOMA-beta
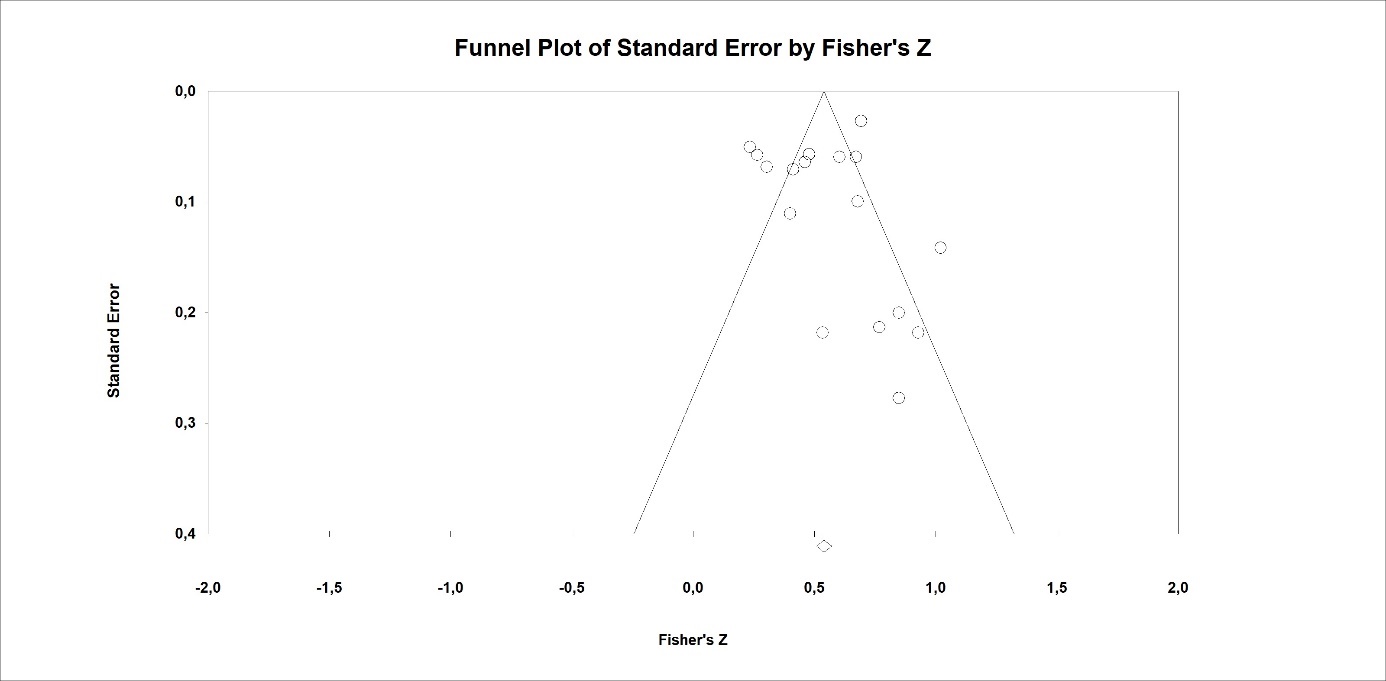

2. IGI(30)


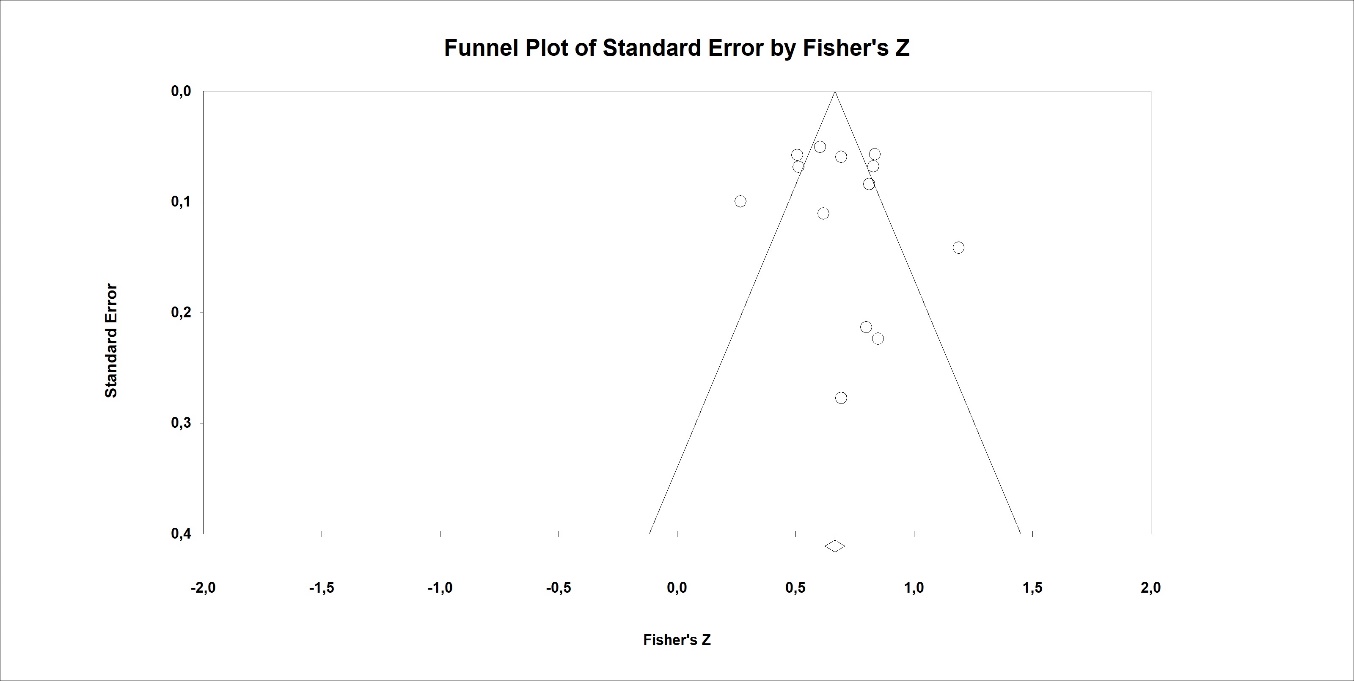


C) Insulin30


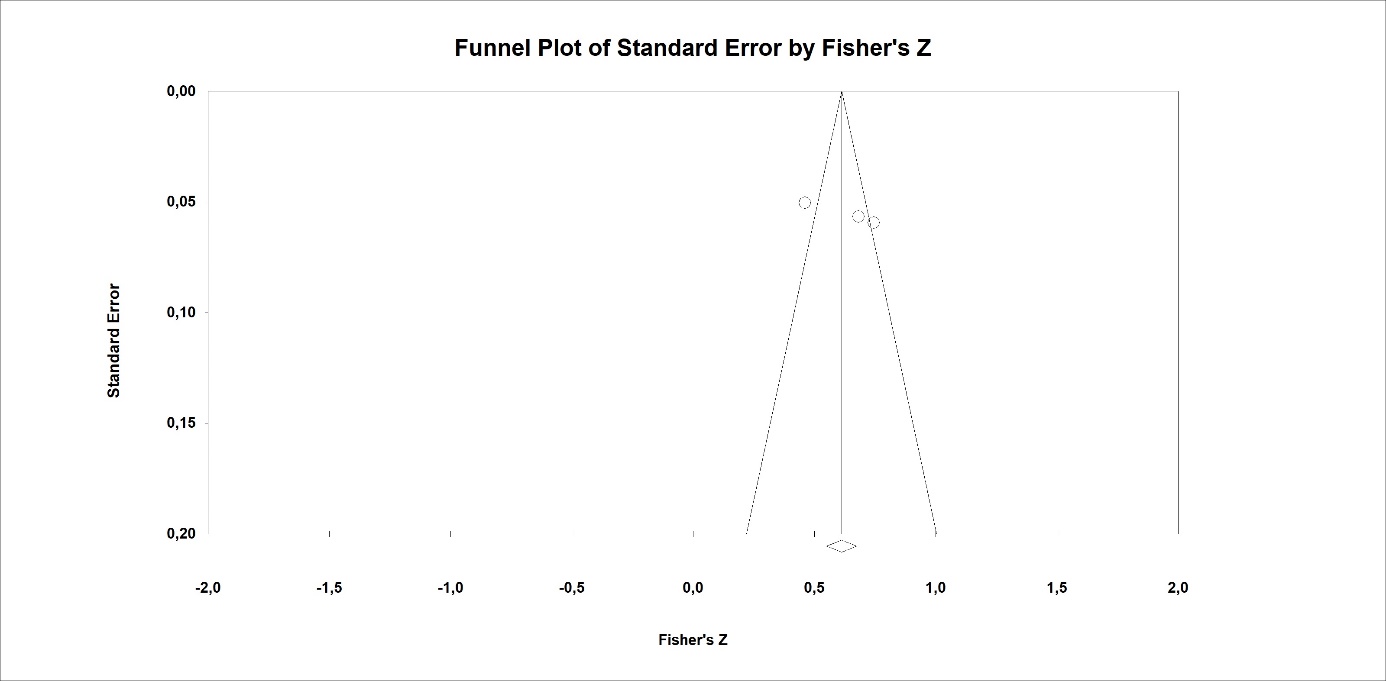


D) Insulin 120


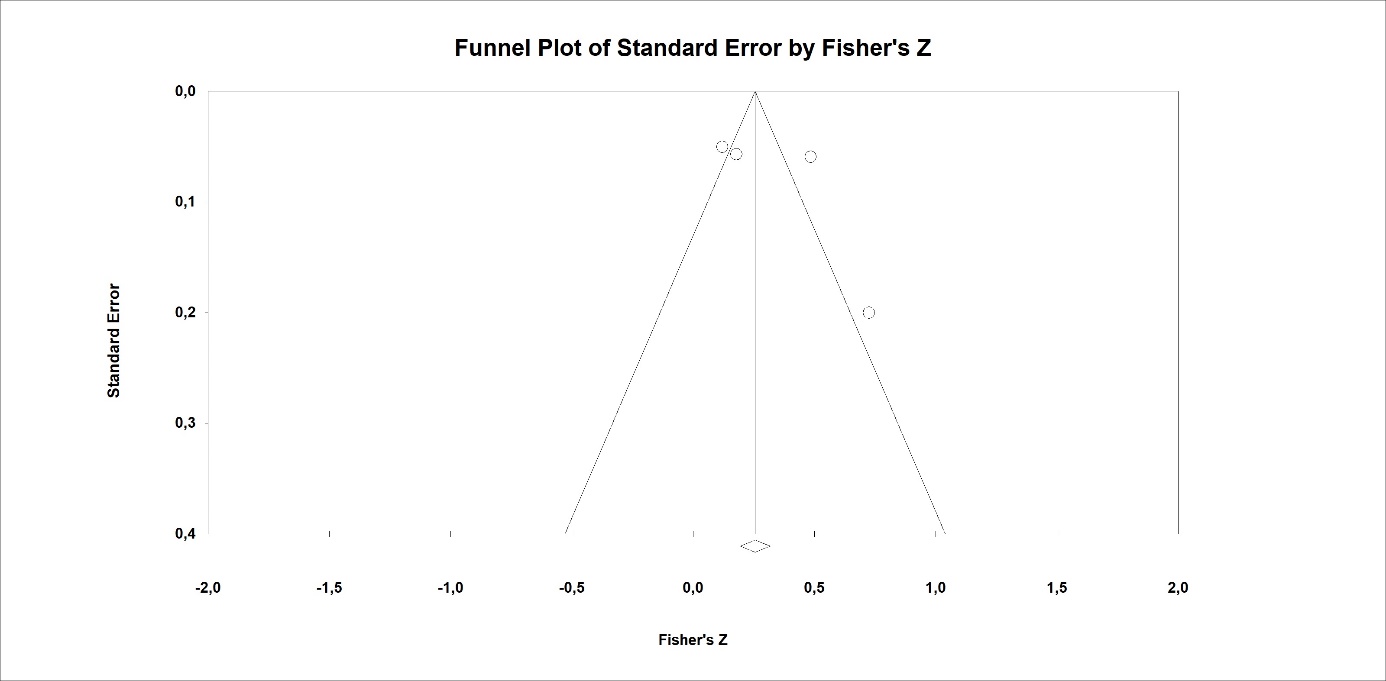


E) AUC30Insulin/AUC30Glucose


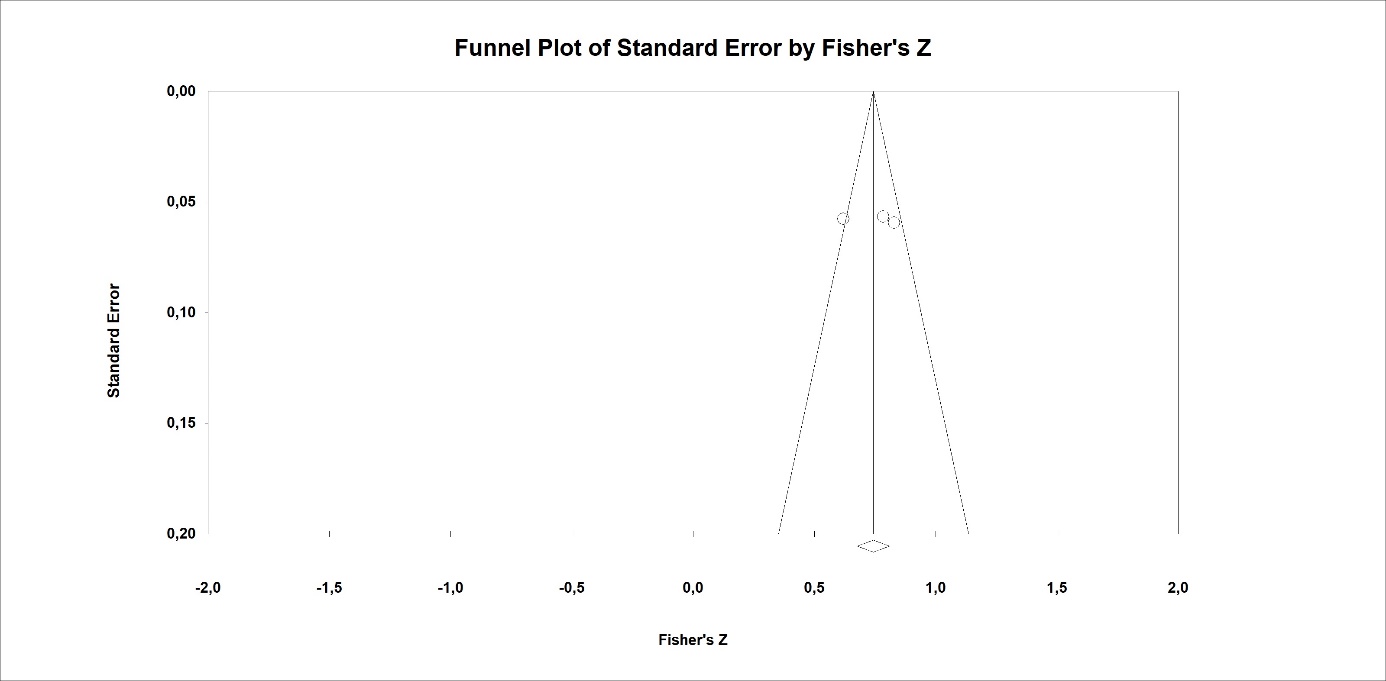


F) Stumvoll’ first-phase secretion


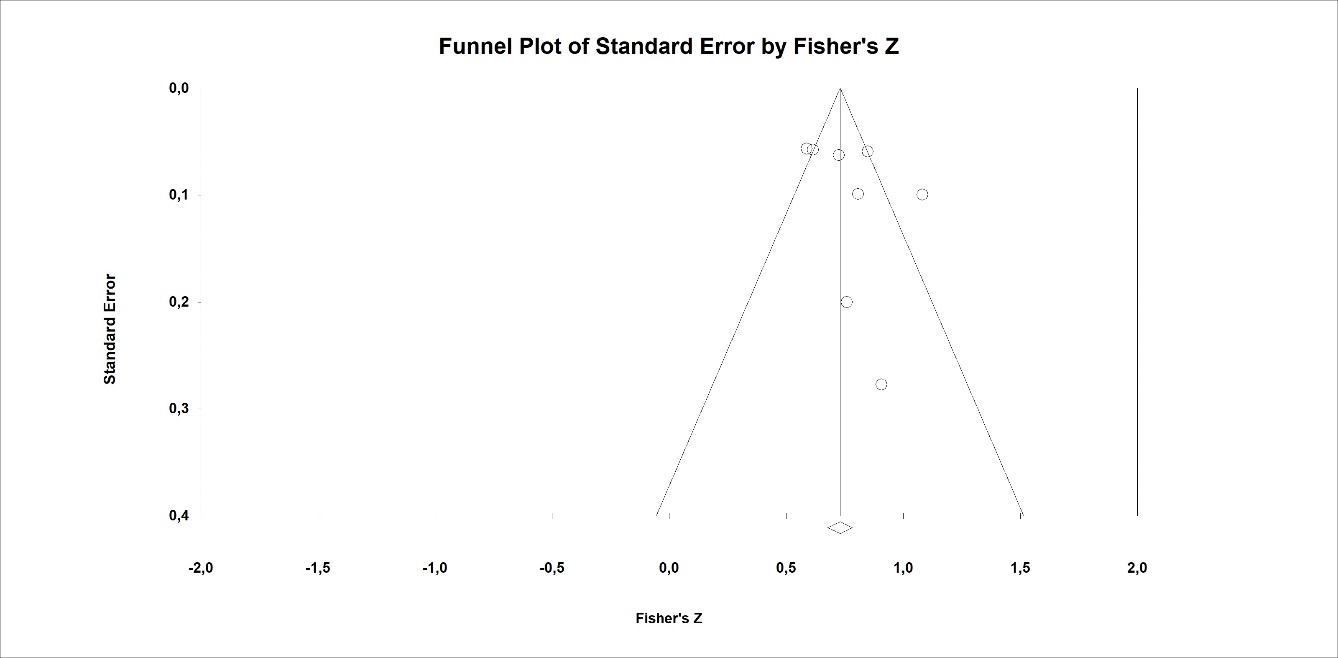
s

G) Stumvoll’s second phase-secretion


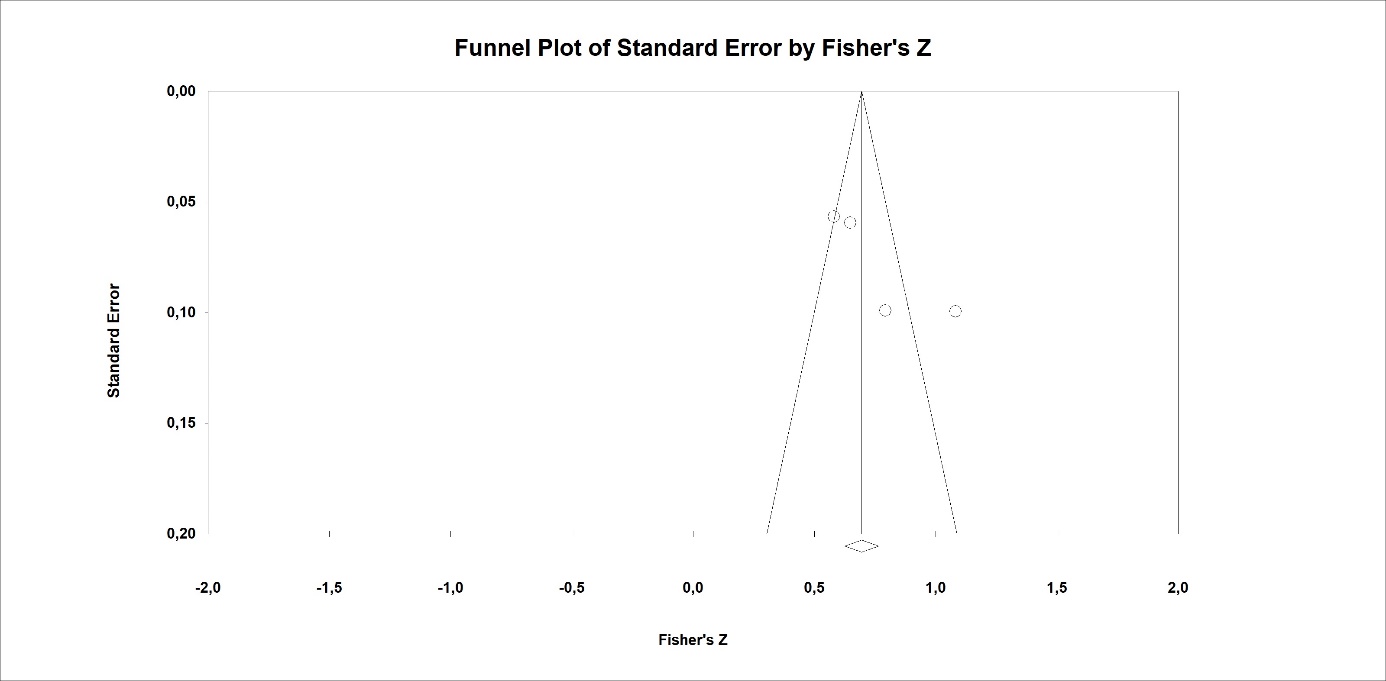


H) AUC120Insulin/AUC120Glucose


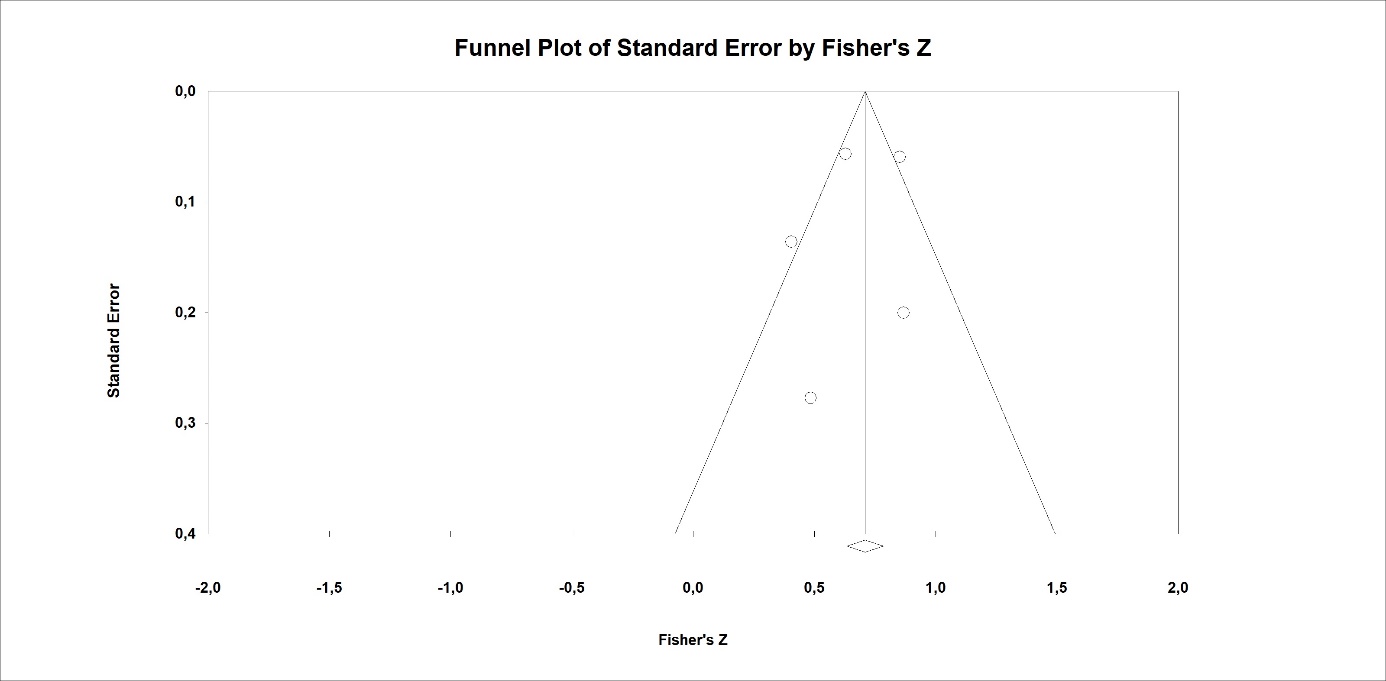


I) BIGTT0-30-120
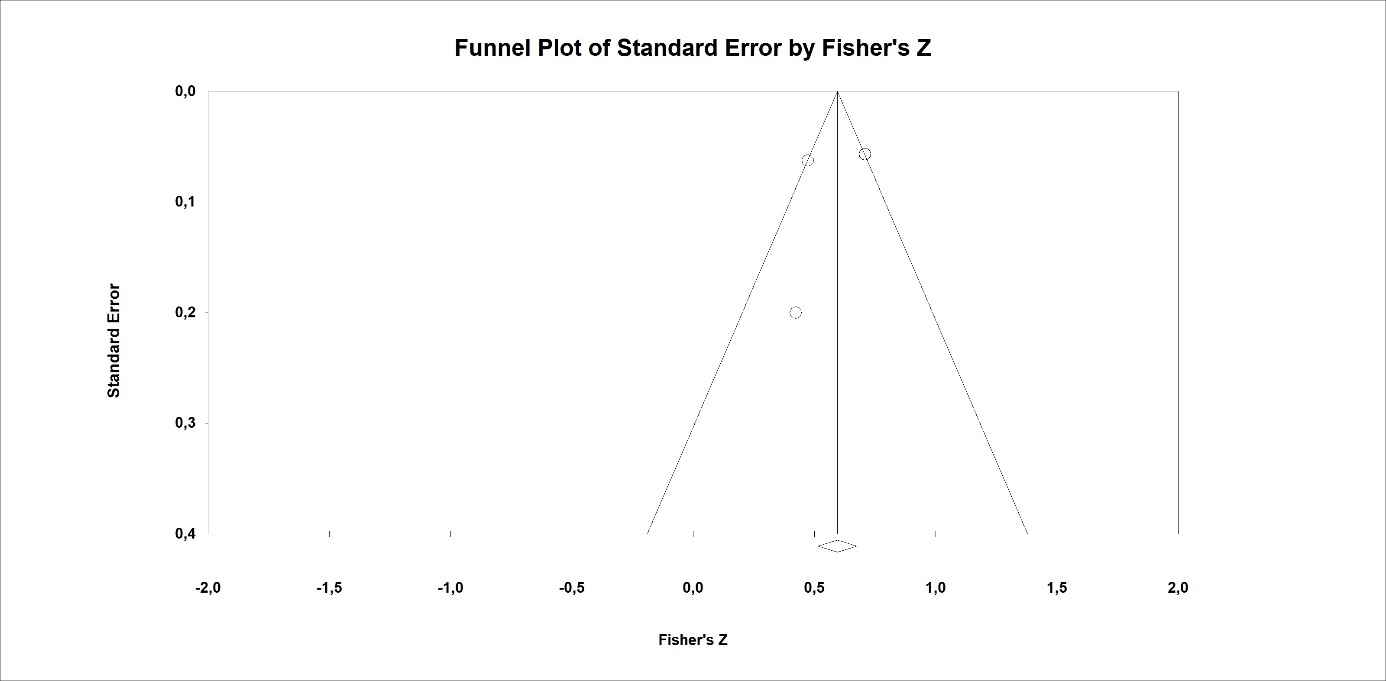


J) BIGTT0-60-120
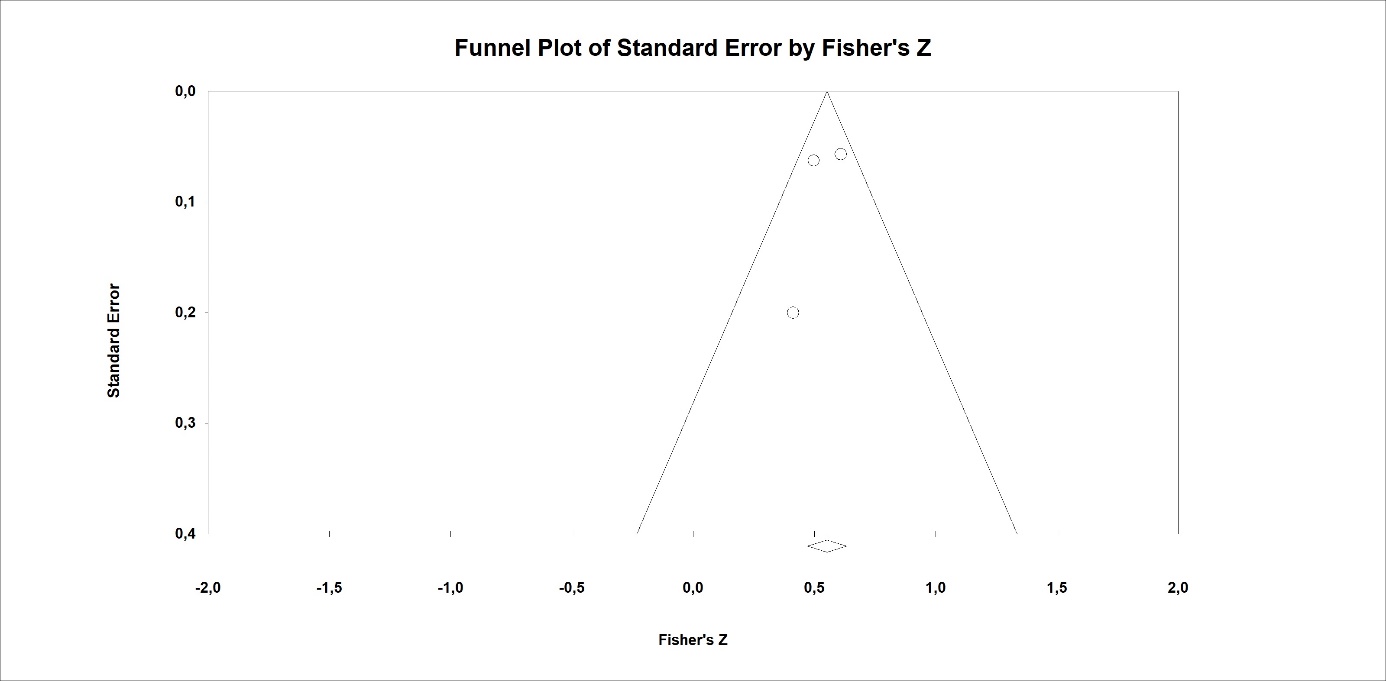
K) Kadowaki


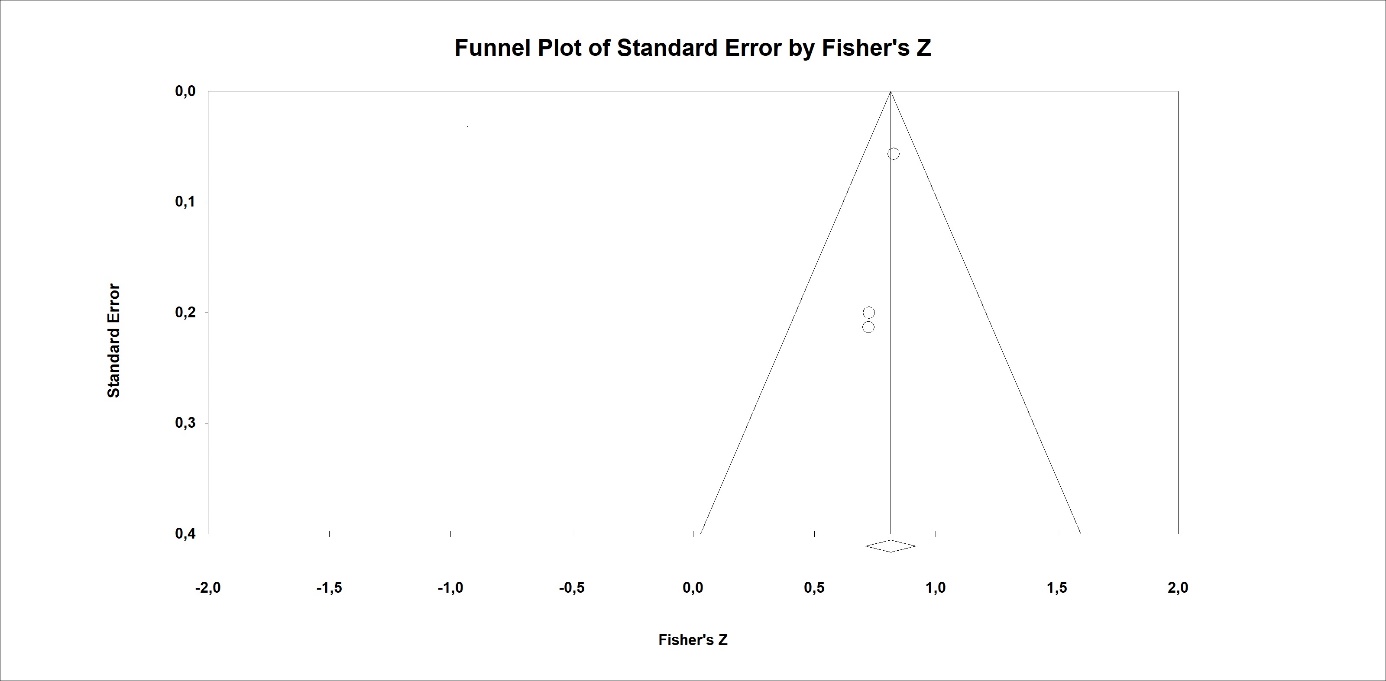


L) Beta-index


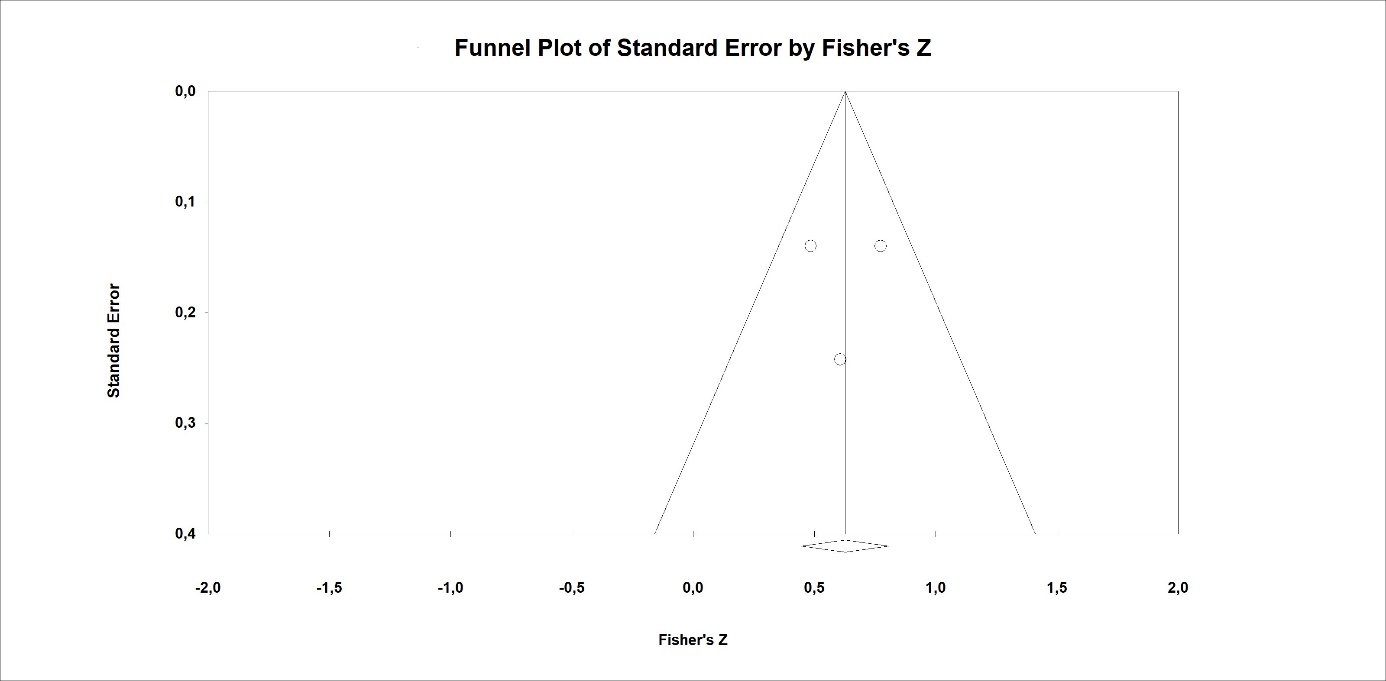


**Suppplementary methods 1** Search phrases

PubMed database

((insulin secretion) OR (beta-cell function) OR (insulin release)) AND ((glucose tolerance test) OR (glucose clamp technique)) AND (human) AND (1947:2020/03/01[pdat])

Web of Science

**((ALL=(insulin secretion)) OR (ALL=(beta-cell function)) OR (ALL=(insulin release))) AND ((ALL=(glucose tolerance)) OR (ALL=(glucose clamp technique))) AND (ALL=(human))** and **English** (Languages)

Timespan: 1945-01-01 to 2020-03-01

Cochrane Central

(insulin secretion OR beta-cell function OR insulin release) AND (glucose tolerance OR glucose clamp technique) AND human

Custom date range: 01/01/1949 to 01/03/2020

Language: English

# References

1. Azzi AS, Cosentino C, Kibanda J, Féry F, Cnop M. OGTT is recommended for glucose homeostasis assessments in Friedreich ataxia. Ann Clin Transl Neurol. 2019;6(1):161-6.

2. Basu R, Breda E, Oberg AL, Powell CC, Dalla Man C, Basu A, et al. Mechanisms of the age-associated deterioration in glucose tolerance: contribution of alterations in insulin secretion, action, and clearance. Diabetes. 2003;52(7):1738-48.

3. Bonadonna RC, Stumvoll M, Fritsche A, Muggeo M, Haring H, Bonora E, et al. Altered homeostatic adaptation of first- and second-phase beta-cell secretion in the offspring of patients with type 2 diabetes: studies with a minimal model to assess beta-cell function. Diabetes. 2003;52(2):470-80.

4. Cretti A, Lehtovirta M, Bonora E, Brunato B, Zenti MG, Tosi F, et al. Assessment of beta-cell function during the oral glucose tolerance test by a minimal model of insulin secretion. Eur J Clin Invest. 2001;31(5):405-16.

5. Chang AM, Smith MJ, Bloem CJ, Galecki AT, Halter JB, Supiano MA. Limitation of the homeostasis model assessment to predict insulin resistance and beta-cell dysfunction in older people. J Clin Endocrinol Metab. 2006;91(2):629-34.

6. Chiu KC, Chuang LM, Yoon C. Comparison of measured and estimated indices of insulin sensitivity and beta cell function: impact of ethnicity on insulin sensitivity and beta cell function in glucose-tolerant and normotensive subjects. J Clin Endocrinol Metab. 2001;86(4):1620-5.

7. Cobelli C, Toffolo GM, Dalla Man C, Campioni M, Denti P, Caumo A, et al. Assessment of beta-cell function in humans, simultaneously with insulin sensitivity and hepatic extraction, from intravenous and oral glucose tests. American Journal of Physiology-Endocrinology and Metabolism. 2007;293(1):E1-E15.

8. Coppack SW, Thursfield V, Dhar H, Hockaday TD. Comparison of indices of islet B-cell function in type 2 diabetes in relation to insulin effectiveness and clinical outcome. Diabet Med. 1991;8(7):629-37.

9. Festa A, Williams K, Hanley AJ, Haffner SM. Beta-cell dysfunction in subjects with impaired glucose tolerance and early type 2 diabetes: comparison of surrogate markers with first-phase insulin secretion from an intravenous glucose tolerance test. Diabetes. 2008;57(6):1638-44.

10. Hammana I, Potvin S, Tardif A, Berthiaume Y, Coderre L, Rabasa-Lhoret R. Validation of insulin secretion indices in cystic fibrosis patients. J Cyst Fibros. 2009;8(6):378-81.

11. Hansen T, Drivsholm T, Urhammer SA, Palacios RT, Volund A, Borch-Johnsen K, et al. The BIGTT test: a novel test for simultaneous measurement of pancreatic beta-cell function, insulin sensitivity, and glucose tolerance. Diabetes Care. 2007;30(2):257-62.

12. Hanson RL, Pratley RE, Bogardus C, Narayan KM, Roumain JM, Imperatore G, et al. Evaluation of simple indices of insulin sensitivity and insulin secretion for use in epidemiologic studies. Am J Epidemiol. 2000;151(2):190-8.

13. Hermans MP, Levy JC, Morris RJ, Turner RC. Comparison of tests of beta-cell function across a range of glucose tolerance from normal to diabetes. Diabetes. 1999;48(9):1779-86.

14. Lehtovirta M, Kaprio J, Groop L, Trombetta M, Bonadonna RC. Heritability of model-derived parameters of beta cell secretion during intravenous and oral glucose tolerance tests: a study of twins. Diabetologia. 2005;48(8):1604-13.

15. Wallace TM, Levy JC, Matthews DR. Use and abuse of HOMA modeling. Diabetes Care. 2004;27(6):1487-95.

16. Levy JC, Rudenski A, Burnett M, Knight R, Matthews DR, Turner RC. Simple empirical assessment of beta-cell function by a constant infusion of glucose test in normal and type 2 (non-insulin-dependent) diabetic subjects. Diabetologia. 1991;34(7):488-99.

17. Maki KC, Kelley KM, Lawless AL, Hubacher RL, Schild AL, Dicklin MR, et al. Validation of insulin sensitivity and secretion indices derived from the liquid meal tolerance test. Diabetes Technol Ther. 2011;13(6):661-6.

18. Mari A, Tura A, Pacini G, Kautzky-Willer A, Ferrannini E. Relationships between insulin secretion after intravenous and oral glucose administration in subjects with glucose tolerance ranging from normal to overt diabetes. Diabet Med. 2008;25(6):671-7.

19. Marini MA, Succurro E, Frontoni S, Mastroianni S, Arturi F, Sciacqua A, et al. Insulin sensitivity, β-cell function, and incretin effect in individuals with elevated 1-hour postload plasma glucose levels. Diabetes Care. 2012;35(4):868-72.

20. Matthews DR, Hosker JP, Rudenski AS, Naylor BA, Treacher DF, Turner RC. Homeostasis model assessment: insulin resistance and beta-cell function from fasting plasma glucose and insulin concentrations in man. Diabetologia. 1985;28(7):412-9.

21. Mohandas C, Bonadonna R, Shojee-Moradie F, Jackson N, Boselli L, Alberti K, et al. Ethnic differences in insulin secretory function between black African and white European men with early type 2 diabetes. Diabetes Obes Metab. 2018;20(7):1678-87.

22. Nijpels G, van der Wal PS, Bouter LM, Heine RJ. Comparison of three methods for the quantification of beta-cell function and insulin sensitivity. Diabetes Res Clin Pract. 1994;26(3):189-95.

23. Okuno Y, Komada H, Sakaguchi K, Nakamura T, Hashimoto N, Hirota Y, et al. Postprandial serum C-peptide to plasma glucose concentration ratio correlates with oral glucose tolerance test- and glucose clamp-based disposition indexes. Metabolism. 2013;62(10):1470-6.

24. Overgaard RV, Jelic K, Karlsson M, Henriksen JE, Madsen H. Mathematical beta cell model for insulin secretion following IVGTT and OGTT. Annals of biomedical engineering. 2006;34(8):1343‐54.

25. Phillips DI, Clark PM, Hales CN, Osmond C. Understanding oral glucose tolerance: comparison of glucose or insulin measurements during the oral glucose tolerance test with specific measurements of insulin resistance and insulin secretion. Diabet Med. 1994;11(3):286-92.

26. Santos JL, Yevenes I, Cataldo LR, Morales M, Galgani J, Arancibia C, et al. Development and assessment of the disposition index based on the oral glucose tolerance test in subjects with different glycaemic status. J Physiol Biochem. 2016;72(2):121-31.

27. Seike M, Saitou T, Kouchi Y, Ohara T, Matsuhisa M, Sakaguchi K, et al. Computational assessment of insulin secretion and insulin sensitivity from 2-h oral glucose tolerance tests for clinical use for type 2 diabetes. J Physiol Sci. 2011;61(4):321-30.

28. Shankar SS, Vella A, Raymond RH, Staten MA, Calle RA, Bergman RN, et al. Standardized Mixed-Meal Tolerance and Arginine Stimulation Tests Provide Reproducible and Complementary Measures of beta-Cell Function: Results From the Foundation for the National Institutes of Health Biomarkers Consortium Investigative Series. Diabetes Care. 2016;39(9):1602-13.

29. Stancáková A, Javorský M, Kuulasmaa T, Haffner SM, Kuusisto J, Laakso M. Changes in insulin sensitivity and insulin release in relation to glycemia and glucose tolerance in 6,414 Finnish men. Diabetes. 2009;58(5):1212-21.

30. Steil GM, Hwu CM, Janowski R, Hariri F, Jinagouda S, Darwin C, et al. Evaluation of insulin sensitivity and beta-cell function indexes obtained from minimal model analysis of a meal tolerance test. Diabetes. 2004;53(5):1201-7.

31. Stumvoll M, Mitrakou A, Pimenta W, Jenssen T, Yki-Jarvinen H, Van Haeften T, et al. Assessment of insulin secretion from the oral glucose tolerance test in white patients with type 2 diabetes. Diabetes Care. 2000;23(9):1440-1.

32. Stumvoll M, Mitrakou A, Pimenta W, Jenssen T, Yki-Jarvinen H, Van Haeften T, et al. Use of the oral glucose tolerance test to assess insulin release and insulin sensitivity. Diabetes Care. 2000;23(3):295-301.

33. Taniguchi A, Nagasaka S, Fukushima M, Sakai M, Nagata I, Doi K, et al. Assessment of insulin sensitivity and insulin secretion from the oral glucose tolerance test in nonobese Japanese type 2 diabetic patients: comparison with minimal-model approach. Diabetes Care. 2000;23(9):1439-40.

34. Tripathy D, Almgren P, Tuomi T, Groop L. Contribution of insulin-stimulated glucose uptake and basal hepatic insulin sensitivity to surrogate measures of insulin sensitivity. Diabetes Care. 2004;27(9):2204-10.

35. Tura A, Kautzky-Willer A, Pacini G. Insulinogenic indices from insulin and C-peptide: comparison of beta-cell function from OGTT and IVGTT. Diabetes Res Clin Pract. 2006;72(3):298-301.

36. van Haeften TW, Dubbeldam S, Zonderland ML, Erkelens DW. Insulin secretion in normal glucose-tolerant relatives of type 2 diabetic subjects. Assessments using hyperglycemic glucose clamps and oral glucose tolerance tests. Diabetes Care. 1998;21(2):278-82.
